# Rebalancing of mitochondrial homeostasis through an NAD^+^-SIRT1 pathway preserves intestinal barrier function in severe malnutrition

**DOI:** 10.1016/j.ebiom.2023.104809

**Published:** 2023-09-20

**Authors:** Catriona Ling, Christian J. Versloot, Matilda E. Arvidsson Kvissberg, Guanlan Hu, Nathan Swain, José M. Horcas-Nieto, Emily Miraglia, Mehakpreet K. Thind, Amber Farooqui, Albert Gerding, Karen van Eunen, Mirjam H. Koster, Niels J. Kloosterhuis, Lijun Chi, YueYing ChenMi, Miriam Langelaar-Makkinje, Celine Bourdon, Jonathan Swann, Marieke Smit, Alain de Bruin, Sameh A. Youssef, Marjon Feenstra, Theo H. van Dijk, Kathrin Thedieck, Johan W. Jonker, Peter K. Kim, Barbara M. Bakker, Robert H.J. Bandsma

**Affiliations:** a Translational Medicine, Peter Gilgan Centre for Research and Learning, The Hospital for Sick Children, Toronto, ON, Canada; b Department of Nutritional Sciences, Faculty of Medicine, University of Toronto, Toronto, ON, Canada; c Department of Pediatrics, Center for Liver, Digestive and Metabolic Diseases, University of Groningen, University Medical Center Groningen, the Netherlands; d Department of Biochemistry, University of Toronto, Toronto, ON, Canada; e Cell Biology Program, Hospital for Sick Children, Toronto, Ontario, Canada; f Department of Laboratory Medicine, University of Groningen, University Medical Center Groningen, the Netherlands; g Faculty of Medicine, School of Human Development and Health, University of Southampton, United Kingdom; h Department of Metabolism, Digestion and Reproduction, Faculty of Medicine, Imperial College London, United Kingdom; i Department of Biomolecular Health Sciences, Dutch Molecular Pathology Centre, Faculty of Veterinary Medicine, Utrecht University, Utrecht, the Netherlands; j Janssen Pharmaceutica Research and Development, 2340, Beerse, Belgium; k Institute of Biochemistry and Center for Molecular Biosciences Innsbruck, University of Innsbruck, Innsbruck, Austria; l Freiburg Materials Research Center (FMF), University Freiburg, Freiburg, Germany; m Division of Gastroenterology, Hepatology, and Nutrition, The Hospital for Sick Children, Toronto, ON, Canada

**Keywords:** Malnutrition, Enteropathy, SIRT1, Autophagy, Mitochondria

## Abstract

**Background:**

The intestine of children with severe malnutrition (SM) shows structural and functional changes that are linked to increased infection and mortality. SM dysregulates the tryptophan-kynurenine pathway, which may impact processes such as SIRT1- and mTORC1-mediated autophagy and mitochondrial homeostasis. Using a mouse and organoid model of SM, we studied the repercussions of these dysregulations on malnutrition enteropathy and the protective capacity of maintaining autophagy activity and mitochondrial health.

**Methods:**

SM was induced through feeding male weanling C57BL/6 mice a low protein diet (LPD) for 14-days. Mice were either treated with the NAD^+^-precursor, nicotinamide; an mTORC1-inhibitor, rapamycin; a SIRT1-activator, resveratrol; or SIRT1-inhibitor, EX-527. Malnutrition enteropathy was induced in enteric organoids through amino-acid deprivation. Features of and pathways to malnutrition enteropathy were examined, including paracellular permeability, nutrient absorption, and autophagic, mitochondrial, and reactive-oxygen-species (ROS) abnormalities.

**Findings:**

LPD-feeding and ensuing low-tryptophan availability led to villus atrophy, nutrient malabsorption, and intestinal barrier dysfunction. In LPD-fed mice, nicotinamide-supplementation was linked to SIRT1-mediated activation of mitophagy, which reduced damaged mitochondria, and improved intestinal barrier function. Inhibition of mTORC1 reduced intestinal barrier dysfunction and nutrient malabsorption. Findings were validated and extended using an organoid model, demonstrating that resolution of mitochondrial ROS resolved barrier dysfunction.

**Interpretation:**

Malnutrition enteropathy arises from a dysregulation of the SIRT1 and mTORC1 pathways, leading to disrupted autophagy, mitochondrial homeostasis, and ROS. Whether nicotinamide-supplementation in children with SM could ameliorate malnutrition enteropathy should be explored in clinical trials.

**Funding:**

This work was supported by the 10.13039/100000865Bill and Melinda Gates Foundation, the 10.13039/501100022221Sickkids Research Institute, the 10.13039/501100000024Canadian Institutes of Health Research, and the 10.13039/501100005075University Medical Center Groningen.

**Research in context**

**Evidence before this study**

Severe malnutrition is linked to almost half of all early childhood deaths, yet, to date, current treatment guidelines are based on minimal scientific evidence and clinical management is not tailored towards the specific organ level dysfunctions with which a child may present. For example, severe malnutrition can induce an intestinal dysfunction that can further exacerbate nutrient malabsorption and poor health. The intestinal barrier is a crucial line of defence, and its’ disruption opens the gates for microbial translocation, and recurrent pathogenic infections. However, no treatments aim to strengthen a compromised intestinal barrier, and this is largely due to the lack of mechanistic understanding that underpins the aetiology.

**Added value of this study**

Our study used a mouse model to reveal mechanistic insights of the intestinal dysfunctions caused by malnutrition. However, our work also specifically tests effects of safe, highly available, inexpensive nutraceutical compounds with plausible value for clinical translation. We have found that severe protein deficiency leads to structural and functional changes of the small intestine, including impaired nutrient absorption and the development of what is known as a “leaky gut” with increased gut permeability. Protein deficiency also disrupts the tryptophan-NAD^+^ pathway resulting in impaired autophagic recycling of mitochondria, the cell’s powerhouse, and mitochondrial homeostasis through the disruption of SIRT1- and mTOR-mediated pathways. We then showed that nicotinamide (i.e., vitamin B_3_), an NAD^+^ precursor, and rapamycin, an mTORC1 inhibitor, protected from disruptions in both mitochondrial homeostasis and functional changes of the small intestine, and these protective changes were found even though conditions of protein deficiency were maintained.

**Implications of all the available evidence**

These findings highlight the possible use of oral administration of nicotinamide (i.e., vitamin B_3_) in modulating tryptophan-NAD^+^-SIRT1 pathway and maintaining mitochondrial homeostasis. Capitalizing on this therapeutic target could aid children with malnutrition in restoring their gut barrier function and reducing bacterial translocation, a specific organ dysfunction commonly occurring in these children. As an adjuvant strategy to current clinical management, the use of either pharmacological or nutraceuticals compounds that can directly improve intestinal barrier function would help to promote survival.

## Introduction

Severe malnutrition (SM) in early childhood remains a major public health concern in low resource geographies, as it contributes significantly to morbidity and mortality in children under five years of age.^1^ Developments in the therapeutic management of SM have improved clinical outcomes,^1^ however, children presenting with diarrhoea and SM are often refractory to treatment and have an increased risk of death.^2^^,^^3^ These children suffer from malnutrition enteropathy, which encompasses alterations in intestinal architecture including villus blunting, intestinal barrier dysfunction^4^, ^5^, ^6^ and nutrient malabsorption.^7^ It has been postulated that the loss of intestinal integrity contributes to clinical deterioration and death through microbial translocation, leading to systemic inflammation and sepsis.^4^^,^^8^^,^^9^ Thus far, the pathophysiological processes involved in the disruption of intestinal homeostasis are unclear and no interventions exist that specifically target the enteropathy in SM.

SM is associated with altered protein metabolism and decreased circulating levels of essential amino acids. In recent studies, low concentrations of serum tryptophan (TRP) and disruption of the TRP-kynurenine pathway have been described in SM.^10^^,^^11^ TRP is an important source of Nicotinamide-Adenine-Dinucleotide (NAD^+^).^12^ The direct involvement of NAD^+^ as a redox carrier in energy metabolism is crucial for cellular function. NAD^+^ also acts as a co-substrate in enzymatic reactions whereby NAD^+^ is cleaved to form nicotinamide (NAM). Subsequently, through the NAD^+^-salvage pathway, NAM is regenerated into NAD^+^. Sirtuin-1 (SIRT1) is an NAD^+^-consuming enzyme that controls cellular metabolic homeostasis through a variety of pathways, including through regulation of autophagy through the deacetylation of effector proteins and mTOR inhibition,^13^^,^^14^ and through mitochondrial biogenesis via activation of peroxisome proliferator-activated receptor-γ coactivator-1α (PPARGC1α). Through these pathways, TRP has been implicated in the pathogenesis of inflammatory bowel disease and other gastrointestinal disorders that involve intestinal barrier dysfunction.^15^, ^16^, ^17^, ^18^, ^19^, ^20^, ^21^ However, a role of the tryptophan-NAD^+^-SIRT1 pathway in malnutrition enteropathy is unknown.

This study aimed to reveal whether the tryptophan-NAD^+^-SIRT1 and autophagy pathways are involved in the aetiology of SM-induced enteropathy and whether modulating these pathways can protect intestinal structure and function. Using mouse and organoid models of malnutrition induced enteropathy,^22^^,^^23^ we discovered that low plasma tryptophan was associated with a reduction in SIRT1 mediated mitochondrial biogenesis and autophagy activity. We demonstrated the therapeutic potential of NAM in ameliorating malnutrition enteropathy and showed that regenerating NAD^+^ through the NAD^+^-salvage pathway with NAM supplementation improved autophagy, intestinal barrier function, and mitochondrial damage. Stimulating autophagy by inhibiting mTORC1 was able to improve intestinal barrier function and lactose absorption, and to preserve mitochondrial morphology. A central role for mitochondrial health in mediating the barrier function in malnutrition was further directly demonstrated using an amino acid deprivation organoid model.

## Methods

### Animals

A mouse model of SM was generated by feeding three-week old weanling mice a low protein diet. Male C57BL/6 J mice (Jackson Laboratory, Bar Harbor, ME, USA) were randomized and placed for two weeks on isocaloric diets, either a low protein diet (LPD, 1% of calories from protein, n = 8) or a normal protein diet (ND, 18% of calories from protein, n = 8) (Supplementary Fig. S1a and b) (Envigo Teklad Diets, Madison, WI, USA). An additional group of mice were fed the LPD and received nicotinamide supplementation (LPD + NAM, n = 8) (Sigma Aldrich, MO, USA) from day seven to day 14 through the drinking water at 0.8 g/L for a final dose of 160 mg/kg of bodyweight (BW).^24^^,^^25^ Only one sex of mice was used for this study to eliminate growth variability. No animals were excluded from analysis. To control for dietary vitamin B3, diets were deficient in niacin, which was titrated back into the drinking water at an adequate dose of 15 mg/kg BW for the ND or a deficient dose 5 mg/kg BW for the LPD. Investigators were not blinded to treatment group. Mice were housed in a light-controlled facility (12 h day night cycles) with a toy for enrichment and *ad libitum* access to water and irradiated diet. The animals were monitored daily by an animal facility technician and by an experimenter for health and humane endpoint, which was 25% body weight loss. All interventions were performed in the day cycle. The primary outcome was intestinal barrier function, and sample size was accordingly calculated using historical data as described previously.^26^ This manuscript adheres to the adheres to standards articulated in the Animal Research: Reporting of In Vivo Experiments (ARRIVE).

### Sirtuin-1 modulation

Additional experiments were performed in which mice were randomized into four groups; (1) a SIRT1 activator group (LPD + RES, n = 6) that received daily treatment with the SIRT1 activator resveratrol via intraperitoneal injections at a dose of 25 mg/kg BW, (2) a SIRT1 inhibitory group that received daily intraperitoneal injections of SIRT-1 inhibitor EX-527 at a dose of 10 mg/kg BW, in addition to nicotinamide supplementation (LPD + EX-527 + NAM, n = 6), (3) a vehicle control group receiving daily IP injections with the vehicle, DMSO (LPD + VEH, n = 6), and (4) a control group (ND, n = 6) (Sigma–Aldrich, St. Louis, MO, USA).^27^^,^^28^ Resveratrol and EX-527 treatments occurred for the final week of the experimental period. Investigators were not blinded to treatment group.

### Rapamycin intervention

Additional experiments were performed in which mice were randomized into three groups: (1) a control group of normal-diet fed animals (ND, n = 6), (2) a set of LPD-fed animals in which mTOR was inhibited with rapamycin via daily intraperitoneal injection at a dose of 6 mg/kg^29^ for the entire experimental period (LPD + RAP, n = 8), (3) and a group of LPD-fed animals received daily injections with the vehicle (LPD + VEH, n = 6). Rapamycin (Sigma–Aldrich, MO, USA) was dissolved in 100% ethanol at 50 mg/ml and diluted in 10% Tween-80 (Sigma–Aldrich). Investigators were not blinded to treatment group.

### *In-vivo* FITC-dextran assay

To assess intestinal permeability, FITC-dextran assays were performed using 4 kDa Fluorescein isothiocyanate (FITC)-dextran (Sigma–Aldrich) as described before,^30^ with a 4-h-fast and 90-min interval between FITC-dextran administration and blood collection.

### Amino acids analysis

Plasma or organoid culture medium was mixed with equal volumes of internal standard (Norleucine), centrifuged at 14,000 rpm for 5 min and subsequently measured on Biochrom 30+ Amino Acid Analyzer (Biochrom, Cambridge, UK).

### Histology

*Murine:* Formalin-fixed intestinal sections were collected, processed, and stained with haematoxylin and eosin (H&E) as described before.^30^ Villus height and crypt depth were measured as described^30^ and were enumerated using Aperio ImageScope (Leica Biosystems, version 12.4) by two blinded board-certified pathologists (ADB&SAY). Immunofluorescence and imaging were performed for CLD-3, OCCL, and HSP60 (Supplementary Table S3) as described^30^ or using a Zeiss-LSM-980 with Airyscan-2 confocal microscope (Zeiss Inc.). Glutaraldehyde-fixed sections for electron microscopy were post-fixed in osmium tetroxide, dehydrated, embedded in resin, stained with uranyl acetate and lead citrate, and imaged with a FEI Tecnai 20 transmission electron microscope (FEIon Company, Hillsoro, OR, USA) or a Zeiss Supra55 in STEM mode at 29 kV using ATLAS-5 (Fibics, Ottawa, ON, Canada) (with the SickKids imaging facility in Toronto).

*Organoid:* Organoids were collected from culture and fixed in 4% PFA for 45 min at 4 °C and processed for confocal fluorescence microscopy as described.^31^ Staining was performed for Zonulin-1, Phalloidin, and E-Cadherin (antibody information and RRID: Supplementary Table S3). Imaging was performed using Zeiss-710 confocal microscope (Zeiss Inc.).

### RNA isolation and real-time qPCR

RNA preparation and qPCR was performed as described before.^30^ Primers sequences (Integrated DNA technologies Inc., Coralville, IA, USA) are listed in Supplementary Table S1.

### Immunoblotting

*Nicotinamide + SIRT1 modulation experiments:* intestinal epithelial cells were collected through overnight incubation 30 mM EDTA followed by shaking and centrifugation. Protein was isolated through sonication in cell lysis buffer (ThermoFisher) and protease inhibitor (Sigma–Aldrich). 20–40 mg protein was electrophoresed on an SDS-Page gel, protein was transferred onto a PVDF membrane, and subsequent antibody incubations were performed (antibody information and RRID: Supplementary Table S3). *Rapamycin experiments:* intestinal mucosa was scraped on ice immediately after sacrifice. Immunoblotting was performed as described.^32^ Signals were normalized to total protein (Simply Blue™ SafeStain, Thermo Fisher Scientific).

### Intestinal glucose and lactose absorption

After a 4-h fast basal blood glucose was assessed. At time point zero, a bolus of [6-6-^2^H_2_]-glucose (∼1000 μmol per kg, Cambridge Isotope Laboratories, MA, USA) was given intravenously via retro-orbital injection, followed by oral administration of a mixed bolus containing D-glucose (∼2000 μmol per kg) and lactose (∼4000 μmol per kg) of which ∼3% was labelled with tracers, i.e., [U-^13^C]-glucose (Cambridge Isotope Laboratories) and [1-^13^C]-lactose (Omicron Biochemicals, IN, USA), respectively. Blood glucose levels were measured, and blood spots were collected every 10-min for 1 h and at 90-min. Absorption of [U-^13^C]-glucose and lactose-derived [1-^13^C]-glucose was calculated as described.^33^, ^34^, ^35^ All equations are listed in Supplementary Table S2.

### Small intestinal organoid generation and culture

Jejunal crypts were isolated and cultured as described before to generate small intestinal organoids with an “apical in” polarization,^36^^,^^37^ with the exception that the organoids were cultured within Cultrex Reduced Growth Factor Basement Membrane Extract (BME) Type 2 (Bio-techne, Minneapolis, MN, USA) instead of Matrigel (CORNING, Corning, NY, USA). Organoids were cultured within complete culture medium consisting of: AdvDMEM/F12 plus 10 mM HEPES, 1 × GlutaMax and 1% Penicillin- Streptomycin (Gibco), 1 × N-2 Supplement (Invitrogen, CA, USA), 1 × B-27 Supplement (Invitrogen), 1.25 mM N-Acetylcysteine (Sigma Aldrich, MO, USA), 10% RSpondin Conditioned Medium, 50 ng/ml EGF and 100 ng/ml Noggin (Peprotech, NJ, USA). To generate a SM phenotype,^36^ small intestinal organoids were cultured in complete culture medium for three-days after passage followed by 48 h of culture in amino acid free medium (AA-Deprived) for 48 h. AA-deprived medium was identical to complete culture medium aside for the use of custom-made amino acid free AdvDMEM/F12 (Life Technologies, Carlsbad, CA, USA). Organoids were visualized under an Olympus CKX41 microscope.

### Polarity reversal of intestinal organoids

“Apical Out” organoids were generated as described before, with minor modifications.^38^ After passage, the organoids were cultured in complete culture medium, as described above, with the addition of 100 ng/ml WNT3-a (Peprotech, NJ, USA) to generate a cystic morphology. Organoids were placed in suspension^38^ after 4–5 days of culture for 72 h, at which point apical out polarity was confirmed via fluorescence microscopy. The organoids were then cultured in complete culture medium, AA-deprived culture medium, AA-deprived +20 μM MitoTempo for 72 h, or AA-deprived culture medium for 72 h with 5 mM NAM treatment for the final 24 h.

### High resolution respirometry

Organoid respiration was measured using a two-channel high-resolution Oroboros Oxygraph-2 k (Oroboros, Innsbruck, Austria) as previously described.^36^ Once in the chamber, organoids were permeabilized with digitonin then given 1 mM ADP. Coupled respiration was achieved by addition of the substrates pyruvate (2 mM) plus malate (2 mM) (State 3). Next, leak respiration (state 4) was determined using oligomycin to block ATP synthase. Finally, 1.5 μM FCCP was added to study the oxygen consumption during uncoupled respiration (State U). Oxygen consumption rates were normalized to cell number.

### Mitochondrial superoxide measurement

Mitochondrial superoxide was quantified in apical out organoids using Mitochondrial Superoxide Assay Kit as per manufacturer’s instructions (ABCAM, Cambridge, UK). Superoxide signal was normalized to cell number.

### *In vitro* FITC-dextran assay

Apical out organoids were collected at the end of the culture period as described above, washed in phenol-red free DMEM, placed in 2 mg/mL 4 kDa FITC-Dextran, and immediately live imaged using Zeiss-710 confocal microscope (Zeiss Inc.).^38^^,^^39^ Average luminal and background fluorescence was quantified using ImageJ software.

### Statistics

All results are expressed as mean ± standard deviation (SD) or standard error of the mean (SD) or median and inter quartile range (IQR), as indicated. Normality was tested using the Shapiro–Wilk test. Differences were assessed with an unpaired two-tailed Student’s T-test, Mann–Whitney U-test for non-parametric data, repeated measures ANOVA for multiple day body weight measurements, or ordinary one- or two-way ANOVA with the corresponding post hoc multiple comparisons analyses. Statistical analysis was performed with GraphPad Prism Software Version 9.02 (GraphPad Software, San Diego, CA, USA). Statistical significance was given as ∗∗∗p-value < 0.001, ∗∗p-value < 0.01 and ∗p-value < 0.05; NS (not significant).

### Ethics

Animal work in Canada was approved by the Animal Care Committee of The Hospital for Sick Children, Toronto (Animal Use Protocol Number: 1000058060/1000030900). Animal experiments in The Netherlands were approved by the Central Authority for Scientific Procedures on Animals (CCD) of The Netherlands and the University of Groningen Ethical Committee for Animal Experiments (Animal Use Protocol Number: 171504-01-001/3).

### Role of funding source

The funding institutions were not involved in this study in its design, data collection, analysis, interpretation, or reporting.

## Results

### Impact of a low protein diet on growth and the small intestine

**Fig. 1:**
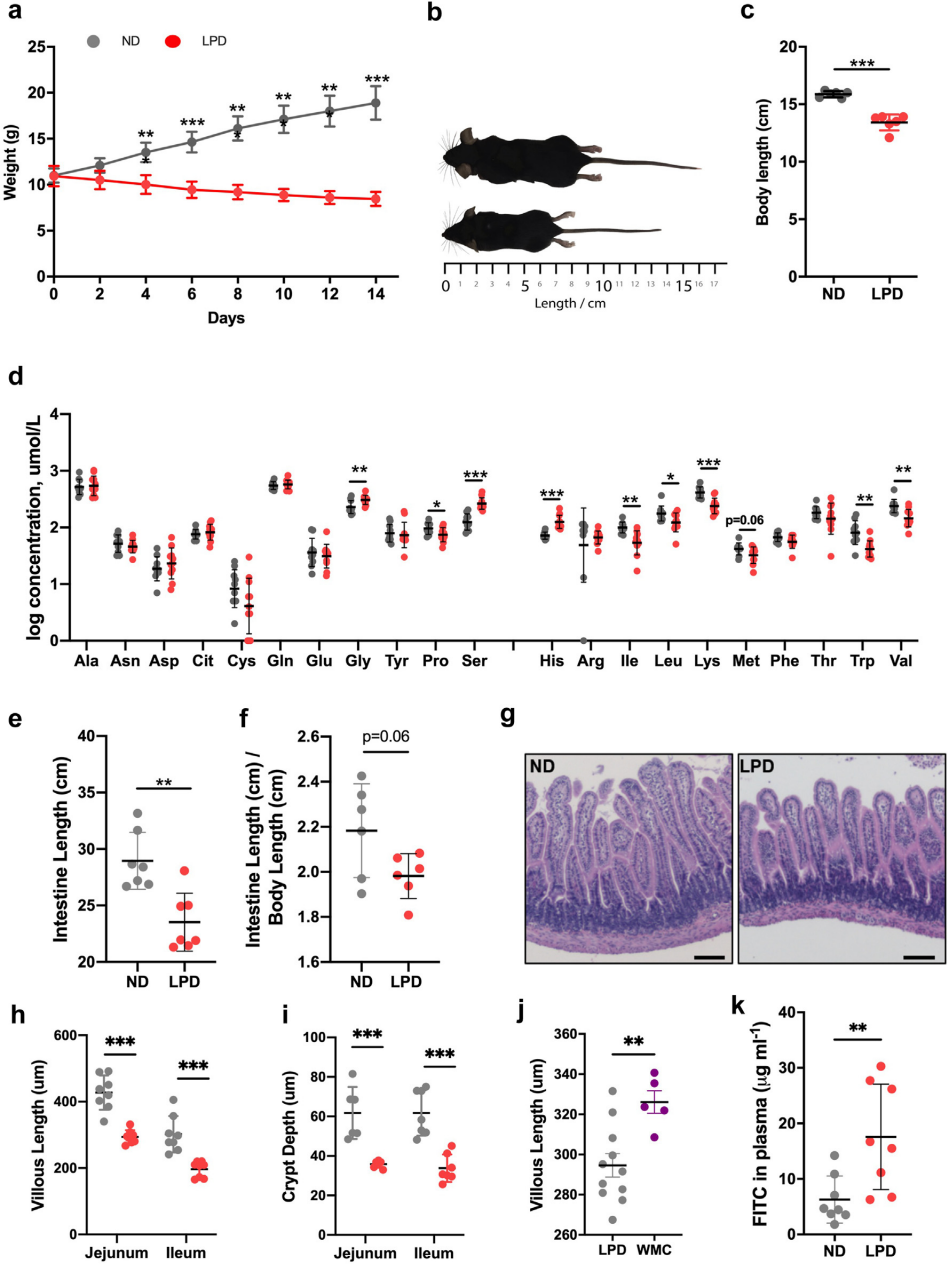
**Assessment of body growth, intestinal architecture and barrier function in weanling mice fed the low protein diet or isocaloric normal diet. (a)** Body weight over 14-days. Individual data points are shown with mean and SD, n = 6 mice per group (repeated measures analysis of variance). **(b)** Representative images of mice in each treatment group. **(c)** Body length measured after 14-days. Data points represent mean SD, n = 6 mice per group. **(d)** Non-essential amino acids (NEAA) and essential amino acids (EAA) were measured in plasma on day 14. Bar graph indicates the mean with SD, n = 10–12 mice per group (two-tailed Student’s T -test on log transformed data). **(e)** Intestine length, and **(f)** intestine length normalized to body length were measured after 14-days. Data points represent mean SD, n = 6 mice per group (Student’s T-test). **(g–i)** Average villus height in jejunum and ileum with representative H&E-stained images of jejunum from each group, n = 8 mice per group. Scale bar, 100 μm. Bar graphs indicate the mean with SD (Mann–Whitney U test). **(j)** Villus height measured in jejunum of LPD-fed mice and weight-matched weanling mice (WMC). **(k)** Concentration of FITC in the plasma measured 1.5 h post oral administration after 14 days. Bars indicate the mean with SD, n = 8 per group, Student’s T-test). (∗p < 0.05, ∗∗p < 0.01, ∗∗∗p < 0.001) (Grey = ND, red = LPD).

We first characterized the impact of 14 days of a low protein diet (LPD) on body growth and the intestine of weanling C57BL/6 J mice. While the diets were isocaloric, mice fed the normal protein diet (ND) gained body weight but those fed the LPD lost weight (Fig. 1a). By day-14, LPD-fed mice had significantly shorter body length indicating marked stunting (Fig. 1b and c). In LPD-fed mice, plasma concentrations of most essential amino acids (EAA) were reduced, including tryptophan, whereas those of EAA histidine and non-essential amino acids glycine and serine were increased (Fig. 1d). The intestine was shorter in LPD-fed mice compared to ND-fed mice and the ratio of intestine to body length trended towards being smaller (p = 0.058, two-tailed Student’s T-test), suggesting that intestinal shortening may have an independent component beyond the simple effects of stunting (Fig. 1e and f). The small intestine (SI) of LPD-fed mice had reduced villus height and crypt depth without histological evidence of inflammation (Fig. 1g–i, Supplementary Fig. S2f–h). The reduced villus height in LPD-fed mice was also found when compared to healthy weanlings of similar body weight (Fig. 1j). To assess whether these structural changes were associated with defects in intestinal barrier function, we administered FITC- dextran via oral gavage and measured levels in the plasma. There was a 2.5-fold increase of FITC in LPD-fed mice compared to ND-fed mice, indicating increased intestinal permeability (Fig. 1k). These data show that the LPD affected body growth, intestinal architecture, and small intestinal permeability.

### Nicotinamide supplementation protects against functional aspects of LPD-induced enteropathy

In both humans and mice, tryptophan is essential and acts as a critical precursor for the *de novo* biogenesis of NAD^+^. Accordingly, we have previously shown that in this mouse model of SM, feeding the LPD leads to a significant reduction in serum NAD^+^ levels.^22^ As a ubiquitous co-factor, NAD^+^ is required for several cellular processes including maintenance of the intestinal barrier. Therefore, we sought to determine whether providing NAM, a precursor of NAD^+^, would protect LPD-fed mice against enteropathy through the stimulation of the NAD^+^ salvage pathway.

**Fig. 2:**
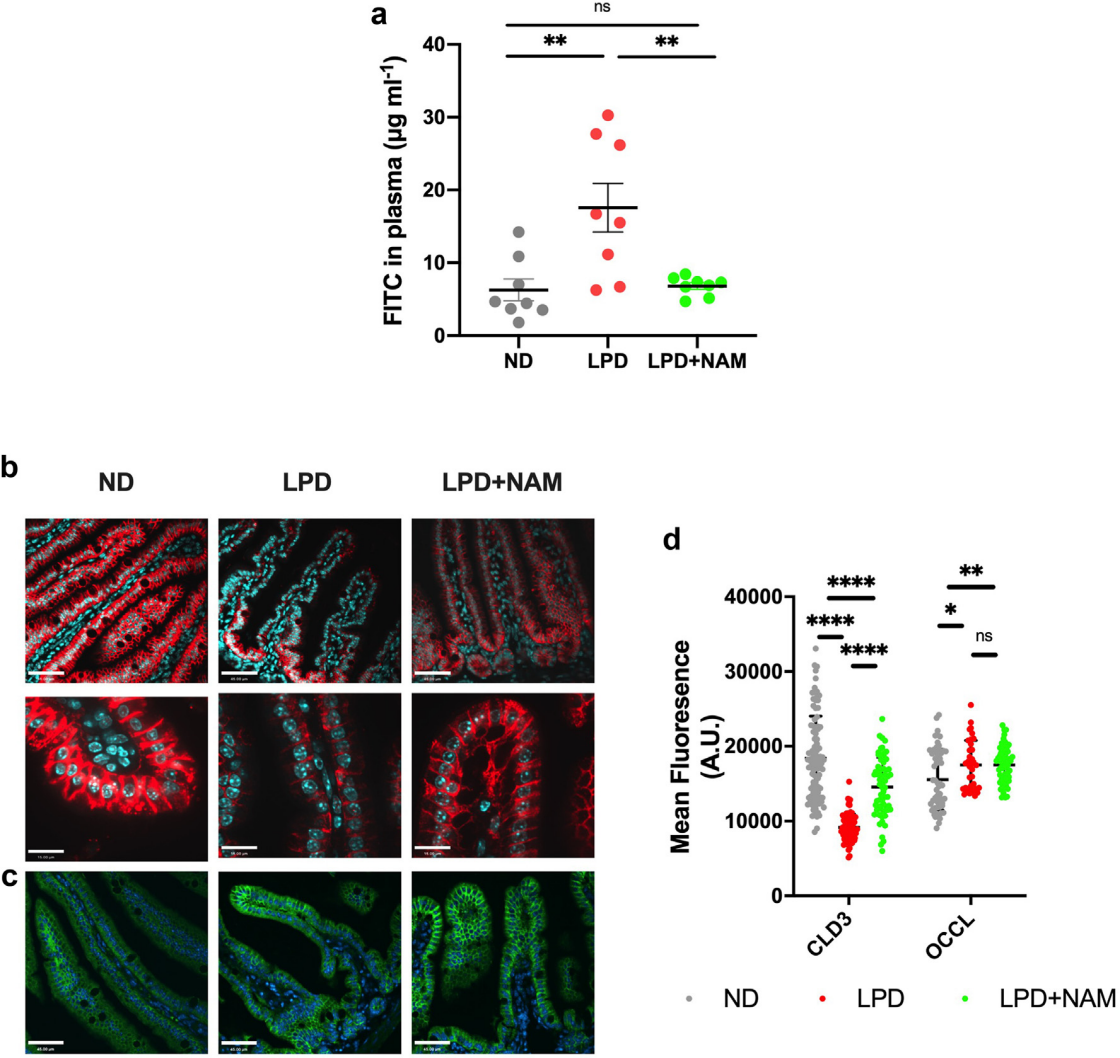
**Supplementation with nicotinamide rescues LPD-induced intestinal barrier dysfunction and loss of tight junction proteins. (a)** Concentration of FITC in the plasma measured 1.5 h-post oral administration on day 14. Bars indicate the mean with SD, n = 8 per group. (Ordinary ANOVA with Tukey’s post-hoc) **(b and c)** Representative immunofluorescent images of CLD-3 (red) and OCCL (green), nuclei counterstained with DAPI (cyan) (scale bar = 45um) **(d)** Quantification of average fluorescence of immunofluorescent staining of CLD-3 and OCCL, bars indicate the mean with SD, n = 3 mice/group, 15–20 image sections/mouse. (Ordinary ANOVA with Tukey’s post-hoc) (∗p < 0.05, ∗∗p < 0.01, ∗∗∗∗p < 0.001).

While NAM supplementation did not affect body weight, intestine length, villus height, or crypt depth of LPD-fed mice (Supplementary Fig. S2a–e), we detected an improvement in the intestinal barrier function, as measured by FITC-dextran plasma levels (Fig. 2a). The integrity of intestinal epithelial cells is maintained by a complex of proteins located at the intercellular junctions forming a barrier called the Tight Junction (TJ).^40^ TJs are composed of combinations of proteins integral to the plasma membrane that include claudin, occludin (OCCL) and junction adhesion proteins. The intestinal epithelial cells of the villus express Claudin-3 (CLD-3), Claudin-4 (CLD-4), Claudin-7 (CLD-7), and Occludin.^41^^,^^42^ In response to LPD, we found that TJ protein CLD-3 was significantly reduced, and CLD-4 trended towards a reduction (p = 0.07, ANOVA with Tukey’s post-hoc comparison), while CLD-7 remained unchanged (Fig. 2b, d, Supplementary Fig. S2i–m). Occludin, however, had a higher protein expression in response to the LPD (Fig. 2c and d). The NAM-induced protection of intestinal barrier function was associated with normalization of CLD-3, but OCCL and CLD-4 protein levels were unchanged (Fig. 2b,d, Supplementary Fig. S2i–m). Together, these data demonstrates that NAM supplementation can prevent increased paracellular permeability in LPD-fed mice likely in part through the maintenance of TJ protein levels.

### The TRP-NAD-SIRT1 pathway is altered in response to a LPD and nicotinamide supplementation

**Fig. 3:**
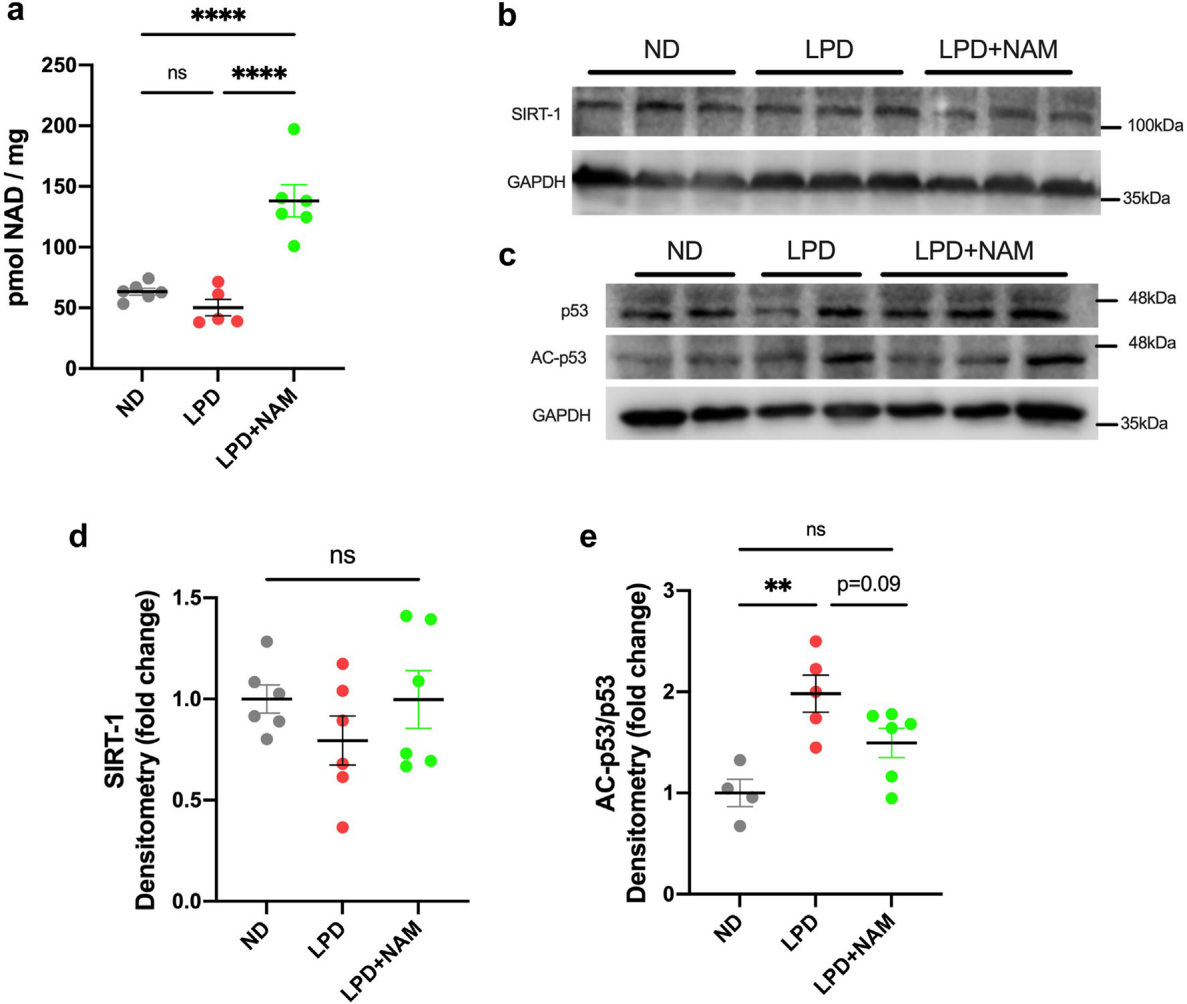
**Feeding a LPD induces alterations in TRP pathway and its downstream target SIRT1. (a)** Small intestinal NAD levels (n = 5–6 per group, Ordinary ANOVA with Tukey’s post-hoc correction) **(b)** Representative immunoblots of SIRT1 and loading control GAPDH **(c)** Representative immunoblots of AC-P53, P53, and loading control GAPDH **(d and e)** densitometry quantification of immunoblots represented as fold change from ND. Bars indicate the mean with SD, n = 6 per group (Ordinary ANOVA with Tukey’s post-hoc comparison to ND). (∗∗p < 0.01∗∗∗∗p < 0.0001).

To understand the mechanism by which NAM supplementation can improve small intestinal barrier function, we explored the possibility that it was increasing small intestinal NAD^+^ levels and improving downstream pathways. Specifically, SIRT1 activation, which regulates mitochondria health and DNA repair^43^ and is NAD^+^ dependent. NAM supplementation improved small intestinal NAD^+^ levels by approximately 2.75-fold compared to the LPD-fed animals (Fig. 3a). Furthermore, despite no significant change in SIRT1 protein levels, the LPD fed animals exhibited a significant reduction in SIRT1 deacetylase activity, as measured by a heightened level of acetylated p53 to total p53 ratio, a commonly measured marker of SIRT1 activity (Fig. 3b–e). The activity of SIRT1 in NAM-supplemented LPD-fed animals trended toward a normalization in SIRT1 activity, as there was a trend towards a reduction in the ratio of acetylated-p53 to total-p53 (p = 0.09, one-way ANOVA with Tukey’s post hoc multiple comparisons, Fig. 3b–e). This indicates that NAM supplementation boosted the cellular levels of NAD^+^ resulting in an improvement in SIRT1 activity.

### Mitochondria of intestinal epithelial cells show signs of damage in LPD-fed animals

**Fig. 4:**
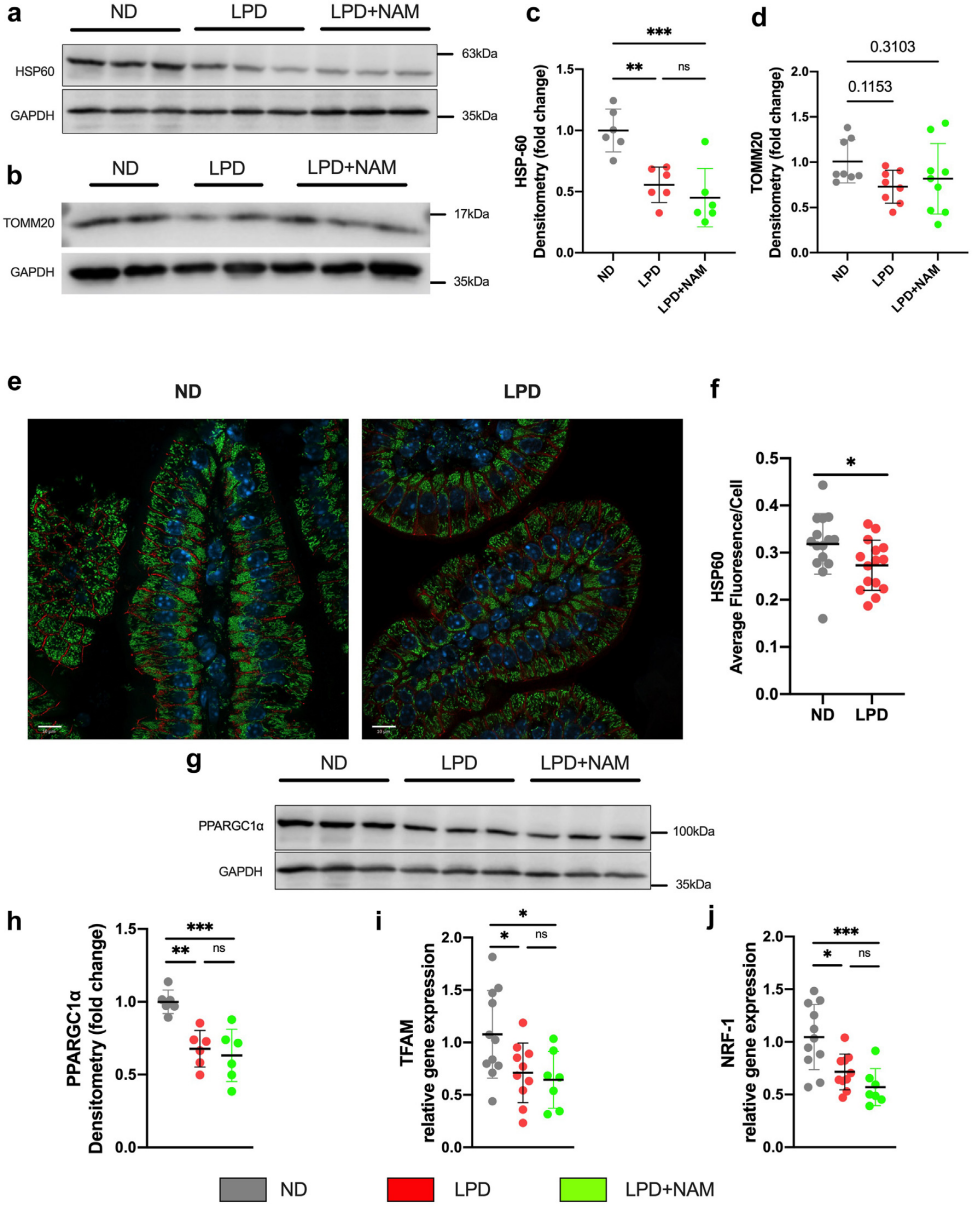
**Feeding LPD induces alterations in TRP pathway and its downstream targets, autophagy and mitochondrial biogenesis. (a and b)** Representative immunoblots of HSP60, TOMM20, GAPDH and **(c and d)** densitometry quantification represented as fold change from ND. Bars indicate the mean with SD, n = 6 per group (Ordinary ANOVA with Tukey’s post-hoc). **(e)** Immunofluorescent imaging of HSP60 (green), membrane marker ECAD (red) and nuclei counterstain DAPI (Cyan) (scalebar = 20um). **(f)** Corresponding quantification of average fluorescence n = 3 mice/group, 15–20 image sections/mouse, individual data points are shown with mean and SD (student’s t-test, ∗p < 0.05) **(g)** Representative immunoblots of PGC-1a and GAPDH and **(h)** densitometry quantification represented as fold change from ND. Bars indicate the mean with SD, n = 6 per group (Ordinary ANOVA with Tukey’s post-hoc). **(i and j)** Gene expression analysis of TFAM and NRF-1, Bars indicate the mean with SD (Ordinary ANOVA with Tukey’s post-hoc) (∗p < 0.05, ∗∗p < 0.01, ∗∗∗p < 0.005).

Altered barrier function and TJ proteins have been linked to impaired mitochondrial function.^13^ We previously reported mitochondrial abnormalities in hepatocytes of rodents during malnutrition-induced hepatic stress.^22^^,^^44^ Thus, given the role of mitochondria in intestinal barrier function,^20^^,^^21^^,^^45^ and known association with SIRT1,^13^^,^^18^^,^^46^ we assessed the intestinal epithelial lining for changes in mitochondrial mass. Mitochondria mass was determined by quantifying immunoblots of intestinal lysates probed for the mitochondrial protein Heat Shock Protein 60 (HSP60) and the Translocase of Outer Mitochondria Membrane 20 (TOMM20). We found HSP60 to be significantly lower in the LPD group compared to ND-fed mice, while TOMM20 was not significantly different it showed a similar trend (Fig. 4a–d). The decrease in HSP60 was also confirmed by immunofluorescence (Fig. 4e and f). Together, this indicates a reduction in mitochondrial abundance in LPD-fed mice. NAM supplementation did not prevent the decrease in HSP60 protein and levels remained unchanged compared to LPD alone (Fig. 4a and b).

To understand the mechanism of this mitochondrial loss, we measured three factors known to regulate mitochondrial bioenergetics and biogenesis: 1) PPARGC1α, a SIRT1-activated transcription co-factor; and the two PPARGC1α regulated genes 2) Nuclear Respiratory Factor 1 (NRF-1); and 3) Transcription Factor A Mitochondria (TFAM).^47^^,^^48^ Immunoblot analyses revealed that protein levels of PPARGC1α were reduced in the LPD-group compared to ND-fed mice (Fig. 4g and h), which was associated with corresponding decreases in TFAM and NRF-1 gene expression (Fig. 4i and j). Thus, the decrease in mitochondrial mass is, at least in part, due to reduced biogenesis. However, mitochondrial biogenesis did not appear to be preserved by NAM treatment in LPD-fed animals as similar levels were seen regardless of supplementation (Fig. 4f–i). This suggests that NAM supplementation in LPD-fed animals was not protective against mitochondrial loss through upregulation of mitochondrial biogenesis.

**Fig. 5:**
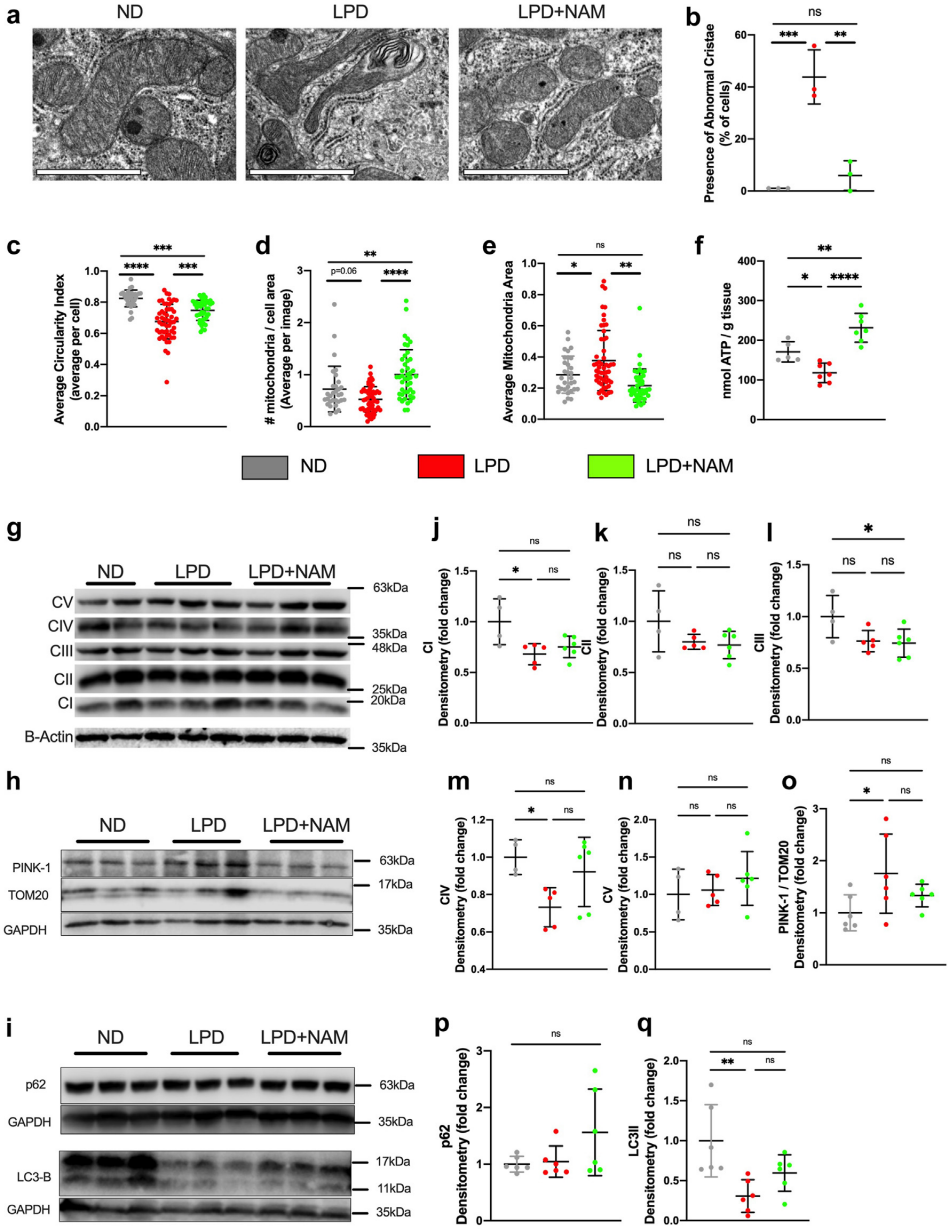
**LPD feeding and nicotinamide supplementation alter mitochondrial complex protein expression and mitochondrial size, quantity, and cristae structures through modulating mitophagy. (a)** Representative TEM images of mouse enterocyte mitochondria (scale bar = 1um). **(b–e)** Quantification of TEM images (N = 3 mice per group with n = 10–20 cells analyzed per mouse (n varying depending on image resolution), individual data points are shown with mean and SD). **(b)** The percent of all cells that contain abnormal cristae structures, (average of cells imaged in n = 3 mice/group). **(c)** The average circularity index of the mitochondria Circularity index is a measurement performed by ImageJ following the calculation *circularity = 4pi(area/perimenter*^*2*^*)*. **(d)** The number of mitochondria per cell area (measured in um^2^). **(e)** The average area of a mitochondria (um^2^), (average of cells imaged in n = 3 mice/group). **(f)** Small intestinal ATP levels (nmol/g tissue, n = 5–7/group). **(g)** Representative immunoblots of Complex I–V and B-Actin, **(h)** representative immunoblots of PINK-1, TOM-20, and GAPDH and **(i)** representative immunoblots of P62, LC3B, and GAPDH. **(j–q)** Densitometry quantification of immunoblots represented as fold change from ND. Bars indicate the mean with SD, n = 6 per group. (All statistical tests shown are: Ordinary ANOVA with Tukey’s post-hoc, ns: p > 0.05, ∗p < 0.05, ∗∗p < 0.01, ∗∗∗p < 0.005, ∗∗∗∗p < 0.001).

Electron microscopy micrograph of the intestinal epithelial cells of the small intestine from the LPD group showed clear morphological changes in their mitochondria consistent with damage, including larger, irregularly shaped mitochondria and severely disrupted cristae structures (Fig. 5a–e). As mitochondrial structure is intricately linked to its function, we measured small intestinal ATP levels and saw a significant reduction in response to LPD-feeding (Fig. 5f). Furthermore, protein expression of mitochondrial complex-I and complex-IV was reduced in response to LPD-feeding (Fig. 5g, j–n), supporting a disruption of the mitochondrial electron transport chain and mitochondrial function. However, the LPD-fed mice with NAM supplementation showed a normalization of mitochondrial number and size, leading to more and smaller mitochondria than in non-supplemented animals (Fig. 5a, d–e). This was despite no improvement in mitochondrial biogenesis and thus suggests a possible increase in mitochondria fission activity. The NAM fed animals also showed an improvement in mitochondrial morphology, including improve cristae structure (Fig. 5a–c), a rescue of ATP levels (p < 0.001), and of complex-IV protein levels compared to LPD only (p = 0.1, one-way ANOVA with Tukey post hoc multiple comparisons) (Fig. 5f and m), suggesting an improvement in mitochondrial function. To further validate the LPD-induced damage to mitochondria, and their protection by NAM treatment, we assayed for PINK1 stabilization, an inductor signal for mitophagy which is the autophagic process that degrades damaged mitochondria. PINK1 is rapidly degraded in healthy mitochondria but accumulates in damaged mitochondria to signal their turnover.^49^ Compared to ND-fed animals, we observed an increase in full length PINK-1 levels in LPD-fed mice, indicating increased in damaged mitochondria (Fig. 5h and o). In turn, NAM supplementation of LPD-fed mice reduced PINK-1 levels (Fig. 5o) suggesting a reduction in the level of damaged mitochondria by NAM.

### LPD feeding leads to signs of reduced intestinal autophagy

Autophagy is a tightly regulated catabolic process that delivers superfluous and damaged cytoplasmic components and organelles to lysosomes for degradation. SIRT1 directly promotes autophagy through deacetylation of several autophagy proteins, including the autophagosome biogenesis factors, ATG5, ATG7 and LC3.^14^^,^^50^ Therefore, we next addressed whether the accumulation of damaged mitochondria may be due to a defect in autophagy. p62 is a protein involved in selective autophagy of protein aggregates and peroxisomes but not mitochondria.^51^ Interestingly, we found unchanged p62 protein levels in both LPD conditions compared to control (Fig. 5i and p). However, the intestine of the LPD-fed mice showed a significant decrease in the autophagosome membrane protein LC3-II, the membrane bound form of LC3, compared to the ND mice (Fig. 5i and q). This suggests reduced autophagy in the intestine in response to the LPD. However, NAM supplementation showed resulted in an LC3II level that was not significantly different from mice fed the normal diet, suggesting a potential improvement in autophagy compared to the LPD alone.

### Rapamycin treatment rescues LPD-induced intestinal barrier dysfunction

**Fig. 6:**
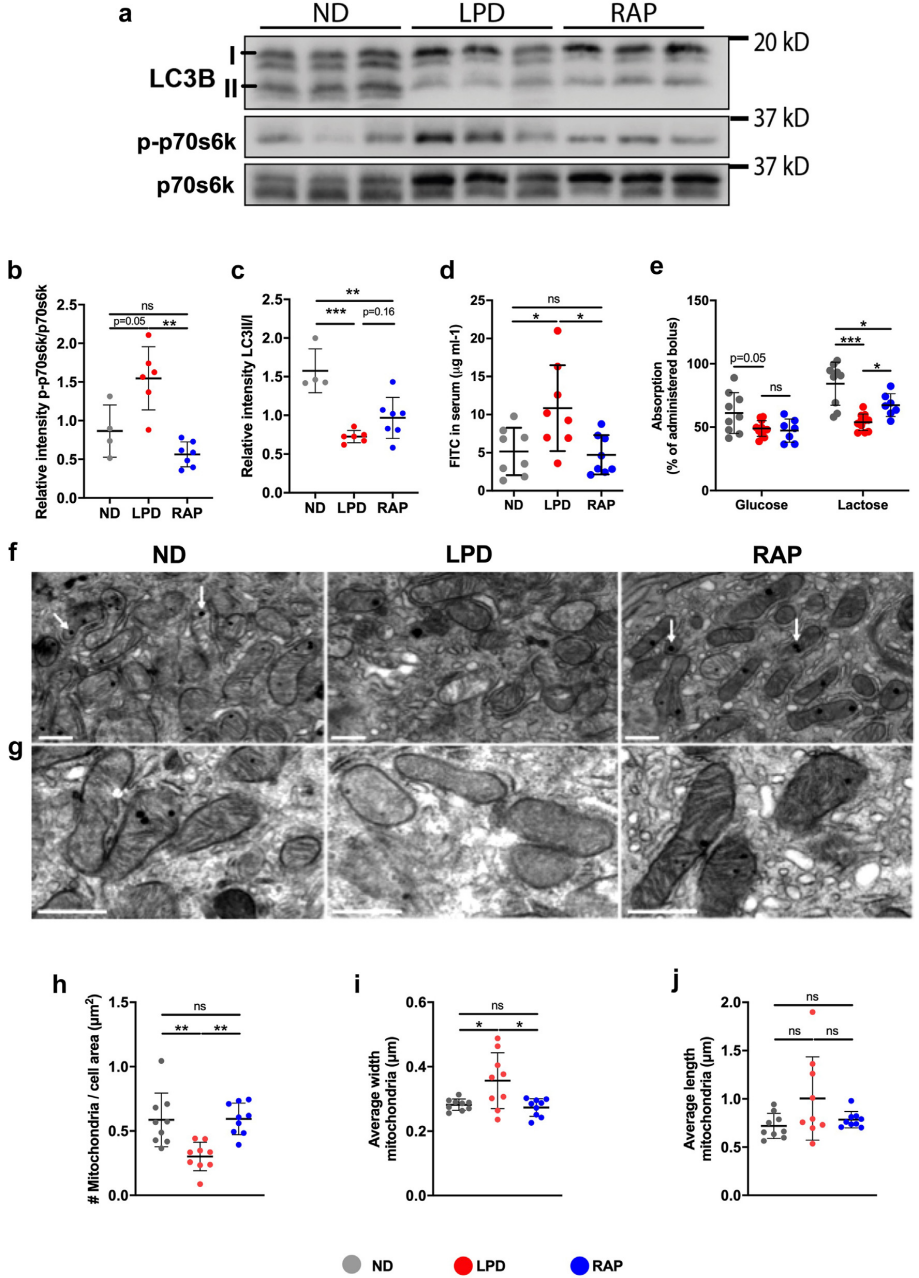
**Autophagy activation via rapamycin treatment improves intestinal function and mitochondrial damage. (a)** Representative immunoblots of LC3B, p70s6k, and p70s6K-pS235/236 and **(b and c)** densitometry quantification normalized to total protein, n = 4–6 mice per group (Individual data points are shown with mean and SD, Two-way ANOVA with Tukey’s post-hoc test). **(d)** Concentration of FITC in the serum measured 1.5 h post oral administration after 14 days. Bars indicate the mean with SD, n = 8 per group (Two-way ANOVA with Tukey’s post-hoc analysis). **(e)** Fractional absorption of glucose and lactose after mice were fed the normal diet (n = 9) or low protein diet (n = 11) or low protein with daily rapamycin i.p., injections for 14 days. Individual data points are shown with mean and SD (Two-way ANOVA with Tukey’s post-hoc analysis). **(f and g)** Representative electron microscopy images of jejunal enterocytes, arrowheads indicating mitochondria (scale bar = 1um). **(h)** Number of mitochondria per cell. **(i)** Average mitochondrial length. **(j)** Average mitochondrial width, n = 9 cells per experimental group (Individual data points are shown with mean and SD, Two-way ANOVA with Tukey’s post-hoc test). (∗p < 0.05, ∗∗p < 0.01, ∗∗∗p < 0.001).

The impairment in autophagy in LPD-fed mice suggest that a defect in autophagy may be contributing to the accumulation of damage mitochondria and a mechanism by which NAM improves mitochondrial function. To test this hypothesis, we examined whether inducing autophagy could mitigate some of the pathogenic phenotypes seen in the intestine of LPD-fed mice. Autophagy is mainly regulated by mTORC1, which inhibits autophagosome initiation.^52^ While acute starvation typically is thought to lead to mTORC1 inhibition, this simplistic understanding may be challenged under the context of prolonged or chronic starvation. Since we have shown SIRT1 inhibition under chronic protein deprivation and SIRT1 is known to inhibit mTORC1,^53^^,^^54^ prolonged starvation may lead to mTORC1 activation and inhibition of autophagosome formation. In support of this hypothesis, we found that the ratio of phosphorylated S6 at serine 235/236 to total S6 was significantly increased in the LPD-fed mice (Fig. 6a and b), indicating mTORC1 activation. To test whether the activation of mTORC1 in the enterocytes of the LPD animals may contribute to the defect in the barrier by inhibiting autophagy, we inhibited mTORC1 using rapamycin. Rapamycin reduced the ratio of phosphorylated S6 total S6 (Fig. 6a and b), indicating reduced mTORC1 activity. Rapamycin treatment led to a trend of increased LC3II/I ratios (p = 0.16, one-way ANOVA with Tukey’s post hoc multiple comparisons) (Fig. 6a and c), suggesting potential improvement in the autophagy pathway. Rapamycin treatment prevented increased paracellular permeability to FITC dextran in LPD-fed mice (Fig. 6d). In contrast, rapamycin did not prevent body weight loss, or the intestinal morphologic changes induced by the low protein diet (Supplementary Fig. S3a–d). Lastly, rapamycin normalized the abundance and morphology of mitochondria to that of the ND-fed mice (Fig. 6f–j), suggesting that increasing autophagy rescues the mitochondrial phenotype.

Monosaccharide and disaccharide malabsorption has been reported in patients with SM.^55^ In line with patient data, we observed that LPD reduced intestinal absorption of lactose and to a lesser extent of glucose (Fig. 6e). To determine whether rapamycin treatment also affected this aspect of intestinal dysfunction, we assessed carbohydrate absorption using ^13^C-labelled lactose and glucose. LPD-fed mice receiving rapamycin showed a partial restoration of intestinal lactose absorption, albeit not entirely to the level of ND-fed mice (Fig. 6e). In contrast, rapamycin did not significantly increase intestinal glucose absorption.

### The effect of nicotinamide supplementation is partially mediated through SIRT1

**Fig. 7:**
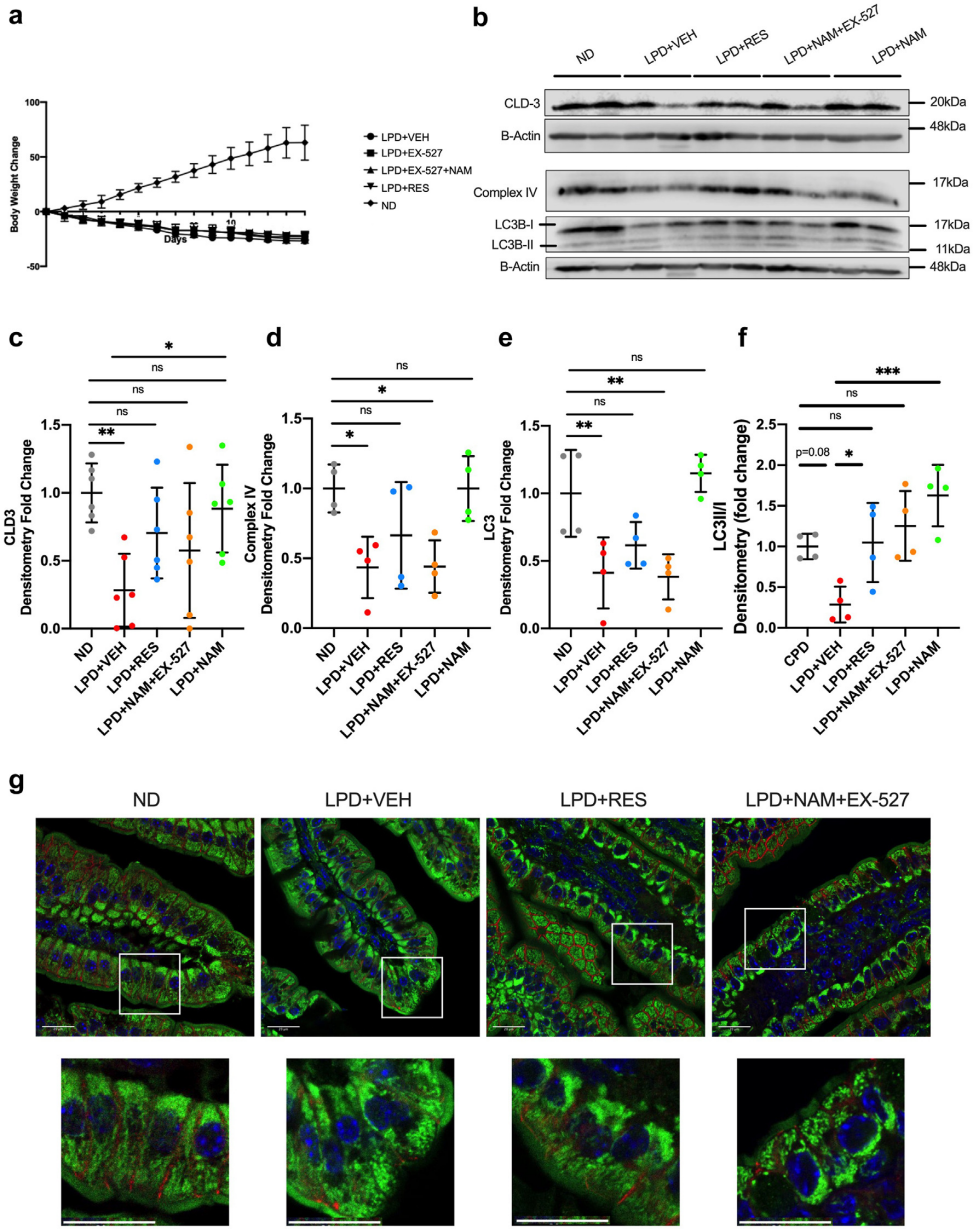
**Nicotinamide-induced alterations in autophagy and mitochondrial complex IV are mediated through a SIRT1 dependent mechanism. (a)** Percent weight change over 14-days (n = 6 per group). **(b)** Immunoblotting of CLD-3, LC3B, ComplexIV, B-Actin and **(c–f)** densitometry quantification of immunoblots represented as fold change from ND. Bars indicate the mean with SD, n = 4–6 per group. NB. GAPDH in for LC3 is the same as for CIV because LC3 and CIV were stripped on the same blot. **(g)** Immunofluorescent imaging of HSP60 (green), membrane marker ECAD (red) and nuclei counterstain DAPI (Cyan) n = 3 mice/group (scalebar = 20um). (All statistical tests shown are: Ordinary ANOVA with Dunnett’s post-hoc, ns: p > 0.05, ∗p < 0.05, ∗∗p < 0.01).

To understand whether the effects of NAM on tight junction proteins and autophagy are mediated through SIRT1 as hypothesized, we directly modulated SIRT1 activity (Supplementary Fig. S4). We observed no improvement in the wasting phenotype in LPD-mice upon SIRT1 activation by resveratrol, and no worsening upon SIRT1 inhibition by EX-527 (Fig. 7a). Resveratrol treatment resulted in a partial recovery of CLD-3 protein level. EX-527 treatment led to similar CLD-3 levels in ND and LPD diet animals (Fig. 7b and c). Upon inhibition of SIRT1, NAM did not affect the levels of LC3II and complex-IV in the LPD group, whereas they were normalized to ND levels in the LPD + NAM only group (Fig. 7b–e). No improvement was observed in LC3-II and complex-IV levels in the LPD + RES animals, suggesting a dependence on sufficient NAD^+^ for the activation of SIRT1. The same trend was seen in regard to mitochondrial morphology, wherein mitochondrial fragmentation appeared to be rescued in the LPD + RES treatment group but the previously seen rescue from NAM supplementation (Fig. 5a and e) was no longer seen under inhibition of SIRT1 with EX-527 (Fig. 7g). These observations give further support to NAM supplementation acts via SIRT1 to improve mitochondrial homeostasis.

### Amino acid deprivation of small intestinal organoids induces features of SM enteropathy

**Fig. 8:**
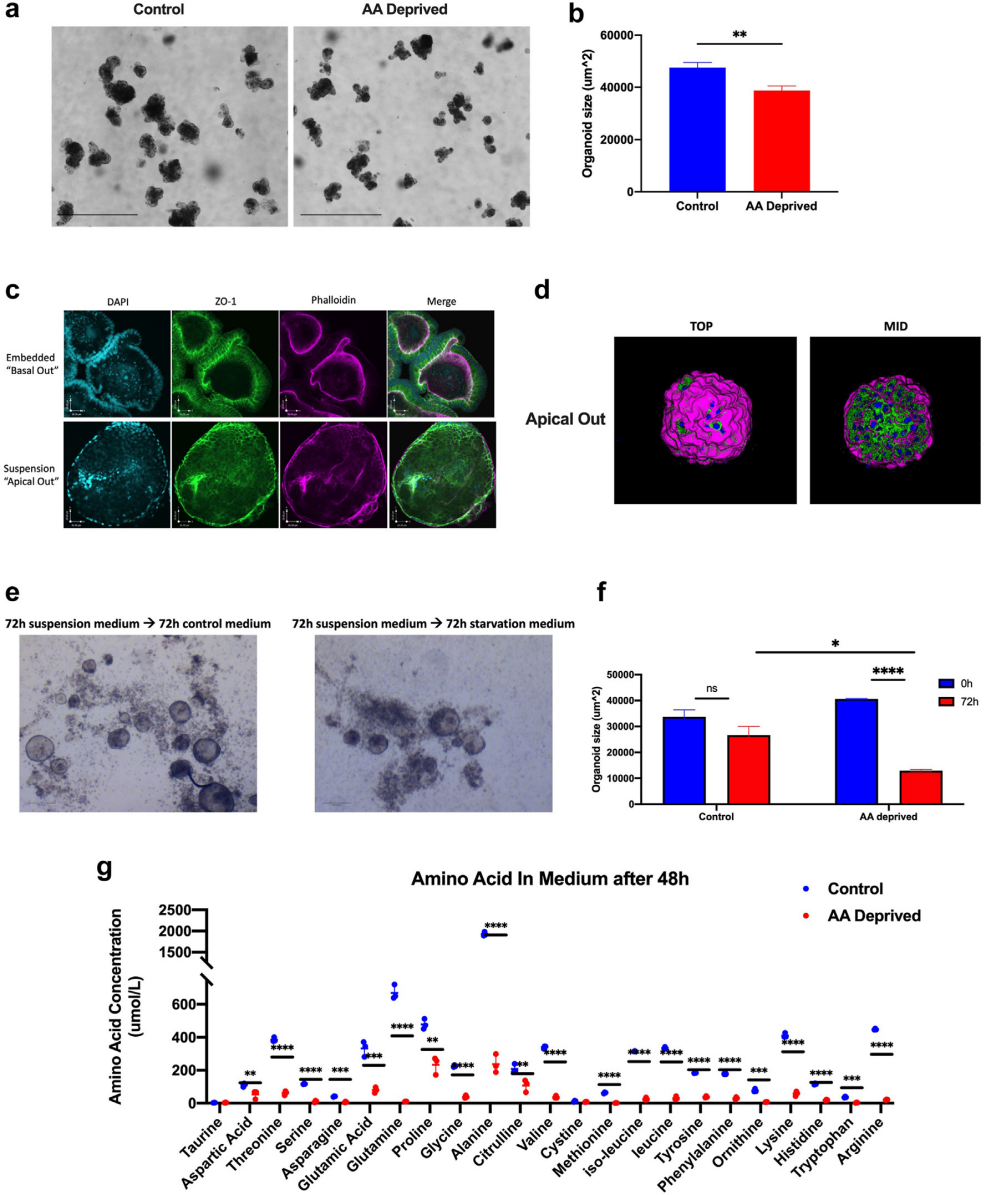
**Amino Acid deprivation of small intestinal organoids induces features of SM enteropathy. (a)** Representative brightfield images of organoids on day 5 (scale bar = 1000uM) **(b)** Average organoid size on day 5 (n = 50–60 organoids in N = 3 biological replicates each, two-tailed Student’s T -test where). **(c)** Representative images of Basal out and Apical out polarization of enteric organoids stained for DAPI (cyan), ZO-1 (green), Phalloidin (magenta) (scale bar = 26uM). **(d)** 3D rendering of exemplary apical out organoid visualized from a top-down view and through the mid-section stained for DAPI (cyan), E-Cadherin (green), Phalloidin (magenta). **(e)** Representative brightfield images of apical out organoids on after 72 h in control or AA-deprived medium (scale bar = 200uM). **(f)** Average organoid size on day 5 (n = 20–30 organoids in N = 3 biological replicates each, two-tailed Student’s T -test where), **(g)** non-essential amino acids (NEAA), and essential amino acids (EAA) were measured in culture medium on day 5. Bar graph indicates the mean with SD, n = 3 biological replicates per group (two-tailed Student’s T-test). (∗p < 0.05, ∗∗p < 0.01, ∗∗∗p < 0.005, ∗∗∗∗p < 0.001).

To better understand the nature and mechanism of the relationship between the protein deprivation induced mitochondrial damage and intestinal barrier dysfunction, and their improvement via NAM supplementation, we used an organoid model of SM enteropathy that we previous developed.^36^ To mimic protein deprivation conditions, jejunal organoids were cultured in an amino acid deficient medium. Compared to organoid grown in complete growth medium, apical-in organoids sere significantly smaller after a period of 48 h in the AA-deprived group (Fig. 8a and b). To better mimic intestinal physiology where the apical is exposed to the outside, we reversed the polarity of the organoids by culturing them in suspension, rendering an apical-out orientation (Fig. 8c and d). Organoid size was also significantly smaller after a period of 72 h in the AA-deprived apical out organoids than in their regular medium controls, suggesting an impairment in growth in either polarization orientation (Fig. 8e and f). All essential and non-essential amino acids were reduced after a 48 h period in the AA-deprived organoids compared to the controls, with the essential amino acids more strongly affected (Fig. 8g).^35^

### Paracellular barrier dysfunction in AA deprived organoids is caused, at least in part, by mitochondrial derived reactive oxygen species

**Fig. 9:**
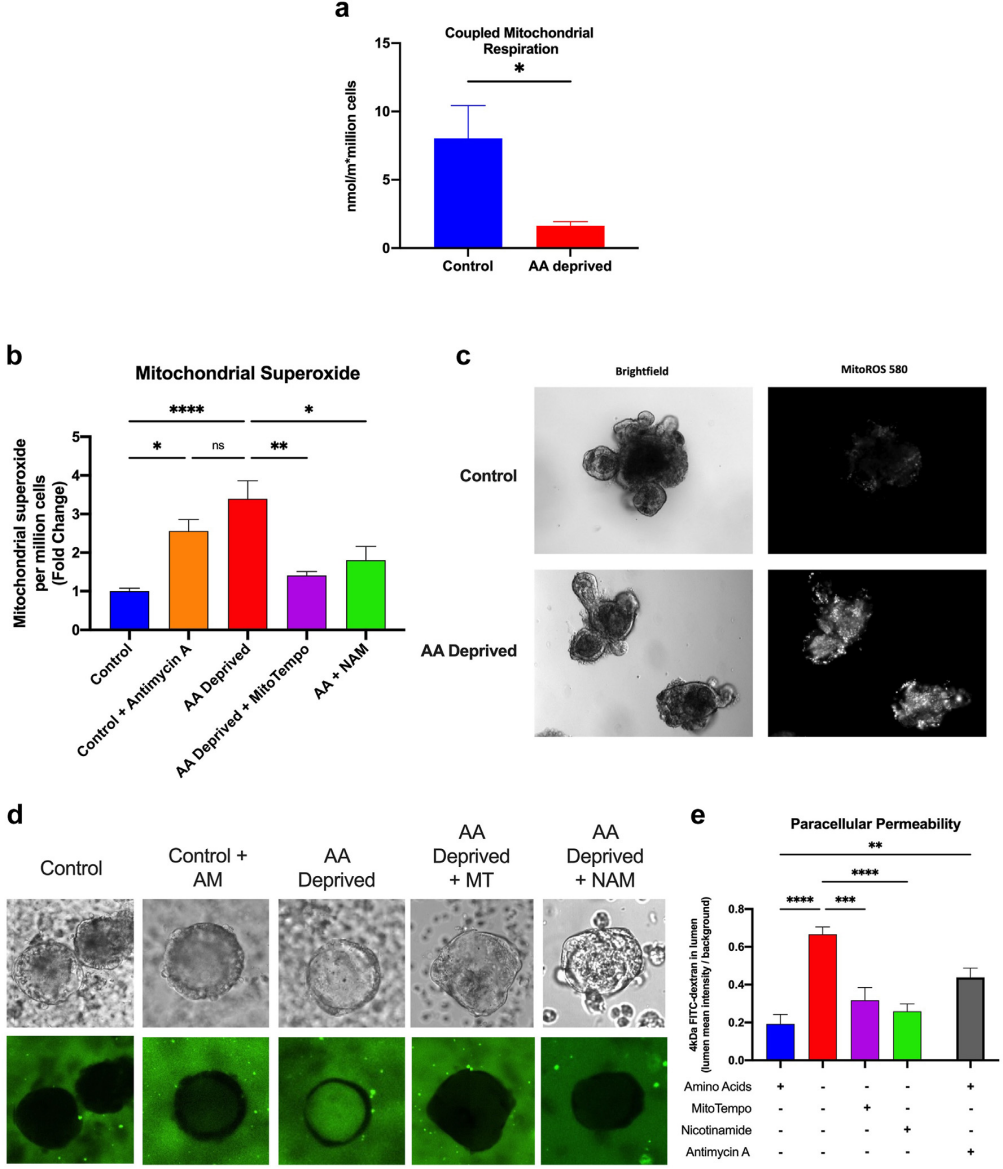
**Paracellular barrier dysfunction in AA deprived organoids is caused, at least in part, by mitochondrial derived reactive oxygen species. (a)** Coupled oxygen consumption measurements in control or amino acid deprived organoids (students two-tailed T-Test). **(b)** Mitochondrial superoxide levels normalized to million cells measured on a fluorescent plate reader (a & b: n = 3 replicates in N = 3 biological replicates each, Ordinary ANOVA with Tukey’s post-hoc). **(c)** Representative fluorescent images of mitochondrial superoxide levels. **(d)** Representative images of the FITC-Dextran Assay in enteric organoids and **(e)** corresponding quantification of FITC-dextran leakage into organoid lumen as lumen intensity/background intensity in (n = 20–40 organoids in N = 3 biological replicates each, Ordinary ANOVA with Tukey’s post-hoc), (ns: p > 0.05, ∗∗p < 0.01, ∗∗p < 0.005, ∗∗∗∗p < 0.001).

We have previously shown that when these organoids are starved of amino acids for 48 h amino acid it results in the loss of the tight junction protein CLD-3 and an increase in paracellular permeability.^36^ Dysfunctional mitochondria have been related to impaired intestinal barrier function through both depletion of energy and reactive oxygen species.^56^, ^57^, ^58^ Therefore, to determine whether an increase in damaged mitochondria contributes to the loss of barrier function during amino acid deprivation, we first examined whether starvation affects mitochondria respiration function of our amino acid deprived organoids. Mitochondrial respiration was measured after 48 h of incubation in control or AA deficient medium. In the presence of pyruvate and malate, coupled respiration was significantly impaired in apical-in organoids deprived of amino acids compared to the controls (Fig. 9a), indicating impaired mitochondrial function in response to AA deprivation. Next, we tested whether the accumulation of damaged mitochondria during prolonged starvation results in an increase in mitochondrially derived reactive oxygen species. Indeed, there was a significantly higher level of mitochondrial superoxide fluorescence in both the apical-in and apical-out organoids grown in amino acid deficient medium compared to those grown in a control medium (Fig. 9b and c). The decrease in superoxide measured when the apical-out organoids were treated with a mitochondria-targeting antioxidant, MitoTempo, supports an increase in oxidative stress in the starved mitochondria (Fig. 9b).

To determine whether the mitochondrial superoxide was causally linked to the impaired intestinal barrier function, we measured paracellular permeability via the leakage of 4-kDa FITC-dextran into the apical-out organoid centre (Fig. 9d). Control, organoids were treated with antimycin A, an inhibitor of ETC complex III that is known to induce superoxide production. They had a 2-fold increase in FITC-dextran leakage into the organoid compared to controls, confirming that mitochondrial superoxide production can damage the paracellular barrier. AA-deprived organoids had a 3-fold increase in paracellular permeability compared to controls, mimicking the impaired intestinal barrier that was observed *in-vivo* (Fig. 9d and e). AA-deprived organoids that were treated with MitoTempo had a significantly lower level of FITC-dextran leakage into the organoid, suggesting that resolution of the excess mitochondrial superoxide can resolve the impairment in paracellular permeability under amino acid deprived conditions (Fig. 9d and e).

Our mice model for SM showed that the NAM treatment improved SIRT1 function, and improved mitochondria health. To test whether NAM treatment can directly improve mitochondria, we treated our organoid model system for amino acid starvation with NAM 24 h before superoxide measurement. We found that NAM resolved excess superoxide at similar levels as those observed with MitoTempo treated organoids (Fig. 9b). Similar, NAM treatment also prevented FITC-dextran diffusion into the apical-out organoids. Together, these studies show that NAM improves intestinal function by improving mitochondrial function.

## Discussion

Our results indicate that feeding a LPD to weanling mice profoundly disrupts the structure and function of the intestine, mimicking the multiple clinically described phenotypes of malnutrition enteropathy. We show that malnutrition-induced intestinal barrier dysfunction is associated with changes in mitochondrial homeostasis resulting in excess mitochondrial derived ROS. The accumulation of damaged mitochondria is partly caused by changes in the NAD-SIRT1-mTORC1-autophagy pathway, which results in the loss of intestinal barrier integrity in SM. Finally, this study demonstrates that NAM and rapamycin can preserve intestinal barrier function and lactose absorption in an animal model of the disease.

Visualization of the small intestinal architecture showed that LPD-feeding induced villus atrophy as seen in SM^4^^,^^59^^,^^60^ and in other animal models of protein restriction.^61^^,^^62^ While we did not explore the cause of the reduction in villus height, findings in humans and other models have led to the hypothesis that severe dietary protein restriction may lead to decreased cellular proliferation, based on a reduction in Ki67 staining in murine small intestinal crypts,^30^ and lower mitotic index in the jejunum of children with SM.^63^ Potential alterations in cellular apoptosis of the villus in this population have not been examined.

Intestinal barrier dysfunction has been reported in children with SM^5^^,^^64^^,^^65^ and it is hypothesized that resulting microbial translocation can lead to a systemic pro-inflammatory state and sepsis.^66^, ^67^, ^68^ We found that LPD-feeding impaired intestinal barrier function, however, we did not explore the possibility of microbial translocation. TJ proteins regulate paracellular permeability and intestinal biopsies from severely malnourished children showed a reduction and alteration in the localization of the TJ proteins claudin-4 and E-cadherin, which provides a plausible molecular basis for disruption of paracellular permeability in SM.^4^ We found that TJ proteins CLD-3 and -4 were reduced in LPD-fed mice, whereas occludin was significantly increased. Previous studies have shown that lacking CLD-3 is sufficient to increase intestinal permeability,^69^ thus loss of CLD-3 and -4 could explain the loss of barrier integrity.^70^ Brown et al. also reported that a LPD in conjunction with microbial exposure induced impaired intestinal barrier function and loss of TJ protein gene expression.^62^ In our LPD model, of the TJ proteins studied, only a selection was reduced, indicating specific transcriptional or post-transcriptional regulation of these proteins in response to LPD. Indeed, CLD-3 and -4 have been reported to be uniquely regulated, independently from other TJ proteins.^71^, ^72^, ^73^ Similarly, other studies have shown a non-uniform response amongst claudin proteins to different insults. For example, a study has shown that in IBD and colon cancer specimens or response to TGF-β signalling, CLD-7 & 4 expression was altered, however not CLD-3.^74^ Tight junction proteins were also differentially regulated in a study where treatment of CACO-2 cells with a flavanol, quercetin, resulted in increased CLD-4 but not CLD-1,-3, or -7 protein expression.^75^ Lastly, in response to a moderate protein deficiency, Brown et al. in their low protein model of malnutrition showed an alteration in intestinal CLD-2 gene expression but not CLD-4, or -15.^62^ It is suggested that occludin may be uniquely regulated through the autophagy pathway.^76^ Therefore, the impairment in autophagy that we observed, may explain the accumulation of occludin.

Intestinal barrier dysfunction was associated with alterations in mitochondrial biogenesis and autophagy. SIRT1 is an NAD^+^ dependent deacetylase that regulates a variety of metabolic and stress responses through lysine deacetylation,^77^, ^78^, ^79^ including mitochondrial biogenesis and autophagy. Modulating the activity of SIRT1 has been found to improve intestinal barrier function in a variety of intestinal models.^18^^,^^80^^,^^81^ In our model, supplementation with the NAD^+^ precursor NAM improved intestinal barrier function with effects through a SIRT1-dependent mechanism. The reduction in SIRT1 protein and activity levels in the LPD group was associated with a reduction in its downstream target PPARGC1α^46^ and subsequent mitochondrial biogenesis.^82^

Our data suggests that the reduction in SIRT1 activity was also associated with reduced autophagy in LPD-fed mice. SIRT1 regulates autophagy via two mechanisms: direct deacetylation of autophagy components, including LC3 and ATG-7,^83^ and indirectly through mTORC1 inhibition.^14^ Supplementation with the NAD^+^ precursor, NAM, partially increased total LC3 in the enterocytes, indicating that LC3II loss may have been through reduced deacetylation by SIRT1. A downstream involvement of mTORC1 is further supported by the intestinal barrier improvement in rapamycin-treated LPD-fed mice. Autophagy has been shown to regulate intestinal barrier function and TJ protein expression, thus may play a role in barrier integrity in our model.^84^, ^85^, ^86^, ^87^

A growing body of evidence suggests that impaired mitochondrial function in epithelial cells can disrupt intestinal barrier integrity, which we hypothesized to be occurring in our model of SM.^88^^,^^89^ The LPD-induced intestinal barrier dysfunction was accompanied by alterations in mitochondrial abundance, morphology, and function. We showed that through NAM supplementation, there may be a partial rebalancing of mitochondrial homeostasis. Damaged mitochondria may have been specifically degraded resulting in a healthier mitochondrial population. Interestingly, more, smaller mitochondria were apparent in the electron microscopy images after NAM supplementation compared to the LPD, despite no changes in mitochondrial protein abundance. This may suggest a potential rebalance of the mitochondrial fission and fusion pathways, which are influenced by NAM and affect the ability of mitophagy to occur.^90^^,^^91^ NAM supplementation also has the potential to rescue mitochondrial function directly through increasing cellular NAD^+^ levels, as sufficient NAD^+^ is necessary for mitochondrial respiration.^92^ Rapamycin also preserved mitochondrial homeostasis, supporting the hypothesis that induction of autophagy improves the mitochondrial phenotype.

Impairments in mitochondrial abundance and function have been shown to relate to IBD- and jaundice-induced intestinal barrier dysfunction.^20^^,^^93^^,^^94^ These studies have suggested that perturbed mitochondrial function may impair the intestinal barrier function via reactive oxygen species or ATP depletion.^20^^,^^93^^,^^94^ Our data in intestinal organoids suggests that while both a depletion of ATP and increased ROS may be impairing the intestinal barrier in our model of SM, resolving mitochondrial superoxide is sufficient to prevent SM induced impairments in paracellular permeability.

A limitation of this study is that the mouse model of SM used lacks an infection component, which is a common presentation in children with SM. Despite this, this model has allowed us to define aspects of SM enteropathy that are caused by a nutritional insult alone, which improves the understanding of the pathophysiology of SM enteropathy. In early stages of in-hospital treatment of severely malnourished children, swift restoration of the intestinal barrier could have the potential to prevent mortality that is related to bacterial translocation. NAM is orally bioavailable and rapidly absorbed across the intestinal epithelium, thus may be used to supplement the stabilization and recovery feeds that children receive in the hospital. While the immune suppressive effect of rapamycin in this patient population with a high burden of infections potentially limits the clinical applicability of this intervention,^95^ NAM has stronger potential to be a therapeutic compound for children with SM, as it has an excellent safety profile. The murine and organoid models presented here can be used to further investigate underlying mechanisms and test potential interventions that could improve intestinal homeostasis in malnourished children.

## Contributors

R.H.J.B., B.M.B, C.L, CJV, P.K.K. were primarily responsible for the study design. C.L. and CJV wrote the manuscript. C.L., C.J.V, M.A.K, G.H, N.S, E.M, M.K.T., J.H.N, A.G., K.E., M.H.K., N.J.K., L.C., Y.Y.C., M.L.M., M.S., M.F., contributed to the conduction of lab experiments. C.L., CJ.V., C.B., E.M., J.K., A.B., S.A.Y., T.H.D, contributed to data analysis. A.F., K.T., J.S., J.W.J., P.K.K., R.H.J.B., and B.M.B. provided expertise, interpreted results, and commented on the manuscript. All authors contributed to editing of the manuscript. All authors read and approved the final version of the manuscript. R.H.J.B, C.L., CJV, B.M.B, had access to and verified all underlying data reported in the manuscript.

## Data sharing statement

Data available upon request. Correspondence and requests for materials should be addressed to R.H.J.B. or B.M.B.

## Declaration of interests

All authors declare no conflicts interests.

## Appendix ASupplementary data

Supplementary Figs. S1–S4 and Tables S1–S3

Western Blot Full gels
