# Supplementary material for: Rebalancing of mitochondrial homeostasis through an NAD+-SIRT1 pathway preserves intestinal barrier function in severe malnutrition
Source: eBioMedicine. 2023 Sep 20;96:104809. doi: 10.1016/j.ebiom.2023.104809 (PMC10520344; doi:10.1016/j.ebiom.2023.104809)
Supplement: Supplementary Figs. S1–S4 and Tables S1–S3 [file mmc1.docx]

**Supplemental Information**

**Table of Contents**

Supplemental Table 1: Primer Sequences 2

Supplemental Table 2. Formulas for calculation of intestinal carbohydrate absorption using stable labeled isotopes 3

Supplemental Table 3. Antibody Information. 4

Supplemental Figure 1. Composition and formulation of experimental diets 5

Supplemental Figure 2. Morphometric alterations in response to nicotinamide supplementation 6

Supplementary figure 3. Morphometric alterations in response to rapamycin treatment 7

Supplementary Figure 4. SIRT1 deacetylase activity altered in response to SIRT1 activation or inhibition 8

| **Gene Name** | **Forward Primer Sequence (5’🡪3’)** | **Reverse Primer Sequence (5’🡪3’)** |
| --- | --- | --- |
| Transcription Factor A, Mitochondrial  *(TFAM)* | CGCAGTGATGTCCGCACAGA | CCTCCCGCCCATGTTGCTTA |
| Nuclear Respiratory Factor 1  *(NRF1)* | CGGGGAATGTGGAGCGTGC | ACCCATGCTGGAAAAACACTTCG |
| TNFa | AGGGTCTGGGCCATAGAACT | CCACCACGCTCTTTCTGTCTAC |
| IL-1β | GGTCAAAGGTTTGGAAGCAG | TGTGAAATGCCACCTTTTGA |
| IL-6 | GTGGCTAAGGACCAAGACCA | GGTTTGCCGAGTAGACCTCA |

***Supplemental Table 1. Primer Sequences***

| **Measure** | **Equation** |
| --- | --- |
| Blood concentrations of glucose tracers (µmol.mL^-1^) | ${\left[ H_{2}^{2}glc \right]_{t}=\left( M_{2} \right)}_{t} x \left[ glc \right]_{t}$  ${\left[ C_{6}^{13}glc \right]_{t}=\left( M_{6} \right)}_{t} x \left[ glc \right]_{t}$  ${\left[ C_{1}^{13}glc \right]_{t}=\left( M_{1} \right)}_{t} x \left[ glc \right]_{t}$ |
| **Equation A.** Concentration curve of IV-administered glucose tracer | ${[H_{2}^{2}glc]}_{t}=C_{0}^{el}e^{-k^{el}.t}-C_{0}^{ab}e^{-k^{ab}.t}$ |
| **Equation B.** Area under the curve | $AUC=\frac{C_{0}^{el}}{k^{el}}-\frac{C_{0}^{ab}}{k^{ab}}$ |
| **Equation C.** Glucose Clearance Rate (GCR) (ml.kg^-1^.min^-1^) | $GCR= \frac{H_{2}^{2}glc}{AUC}$ |
| **Equation D.** Rate of disposal (Rd_t_)  (µmol.kg^-1^.min^-1^) | ${Rd}_{t}=GCR x \left[ A_{x}^{y}glc \right]_{t}$ |
| **Equation E.** Rate of appearance (Ra_t_)  (µmol.kg^-1^.min^-1^) | ${Ra}_{t}={Rd}_{t}+ \frac{d\left[ A_{x}^{y}glc \right]_{t}}{dt} pV$ |
| **Equation F.** Total rate of appearance  (µmol.kg^-1^) | $Ra={Ra}_{t}*t$ |
| **Equation G.** Fractional absorption (%) | $F=\frac{Ra}{bolus}$ |

***Supplemental Table 2.* *Formulas for calculation of intestinal carbohydrate absorption using stable labeled isotopes***

| **Antibody** | **Company** | **Species** | **RRID** |
| --- | --- | --- | --- |
| SIRT1 | Cell Signaling | Mouse | AB_10999470 |
| PPARGC1α (PGC-1α) | Abcam | Rabbit | AB_881987 |
| LC3B | Sigma | Rabbit | AB_796155 |
| HSP60 | Abcam | Rabbit | AB_881444 |
| CLD-3 | Abcam | Rabbit | AB_301648 |
| CLD-4 | Invitrogen | Mouse | *NO RRID (INVITROGEN 32-9400)* |
| CLD-7 | Invitrogen | Rabbit | *NO RRID (INVITROGEN 34-9100)* |
| OCCL | Abcam | Rabbit | AB_2737295 |
| TOMM20 | Santa Cruz | Rabbit | AB_2207533 |
| ECAD | Sigma | Rat | AB_477600 |
| Total OXPHOS Rodent WB Antibody Cocktail   - Complex I (NDUFB8) - Complex II (SDHB) - Complex III (UQCRC2) - Complex IV (MTCO1) - Complex V (ATP5A) | Abcam | Mouse | AB_2629281 |
| Pink-1 | Novus Biologicals | Rabbit | AB_526188 |
| P62 | Novus Biologicals | Rabbit | AB_10011072 |
| GAPDH | Novus Biologicals | Mouse | AB_10077627 |
| S6 Ribosomal Protein | Cell Signaling | Mouse | AB_1129205 |
| Phospho-S6 Ribosomal Protein (Ser235/236) | Cell Signaling | Rabbit | AB_2181035 |
| p53 | Cell Signalling | Mouse | AB_331743 |
| AC-p53 | Cell Signalling | Rabbit | AB_823591 |
| DAPI | Abcam | Rabbit | *NO RRID (ab-228549)* |
| Anti-Rabbit | Invitrogen | Goat | *NO RRID (invitrogen AC27040)* |
| Anti-Mouse | Invitrogen | Goat | *NO RRID (invitrogen A28175)* |

**Supplemental Table 3. Antibody Information.**

**
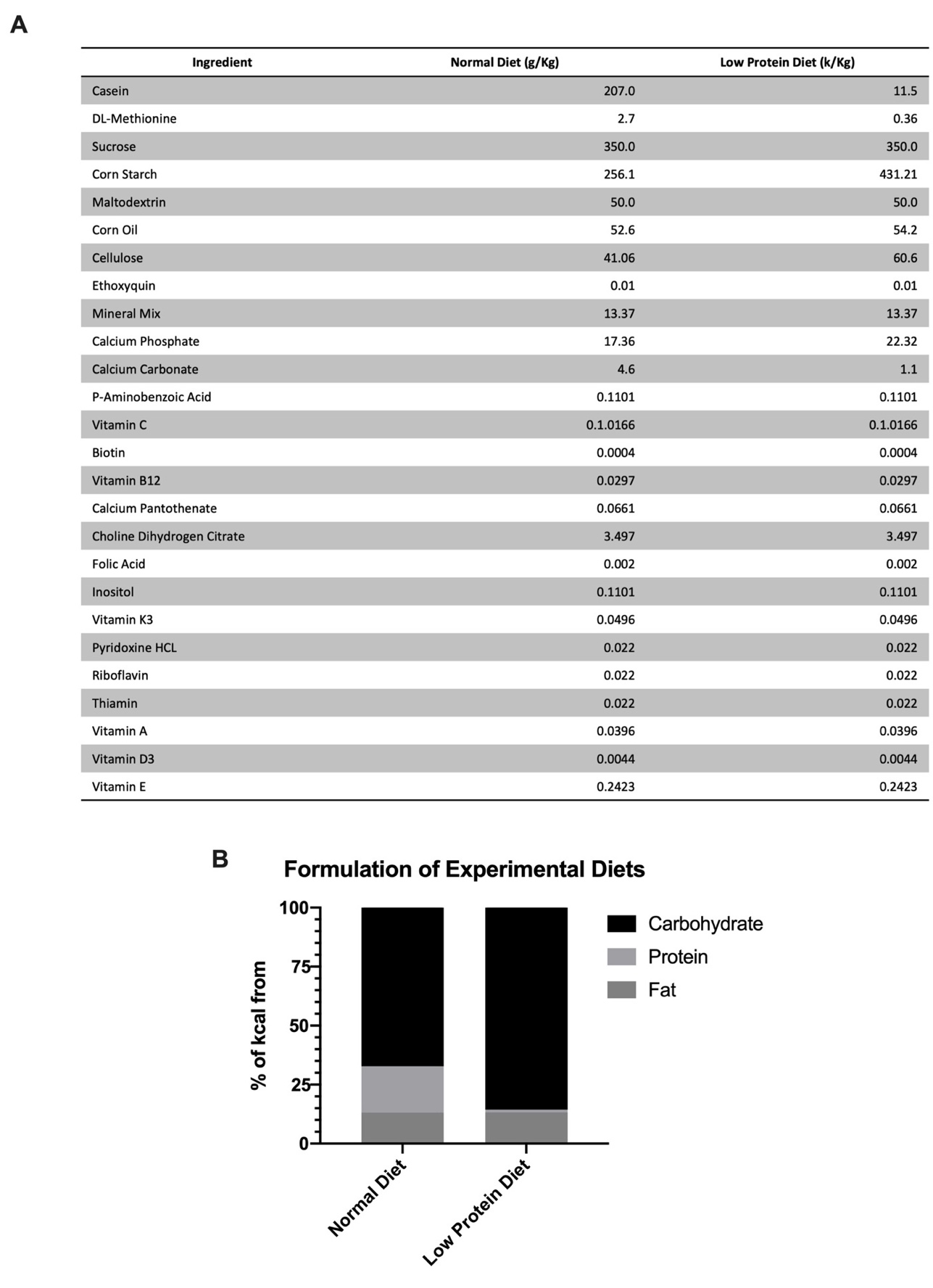
**

**Supplemental Figure 1. Composition and formulation of experimental diets.** (a) Composition of experimental diets. (b) Description of macronutrient composition of diets, shown as %kcal from carbohydrate, protein, and fat.

**
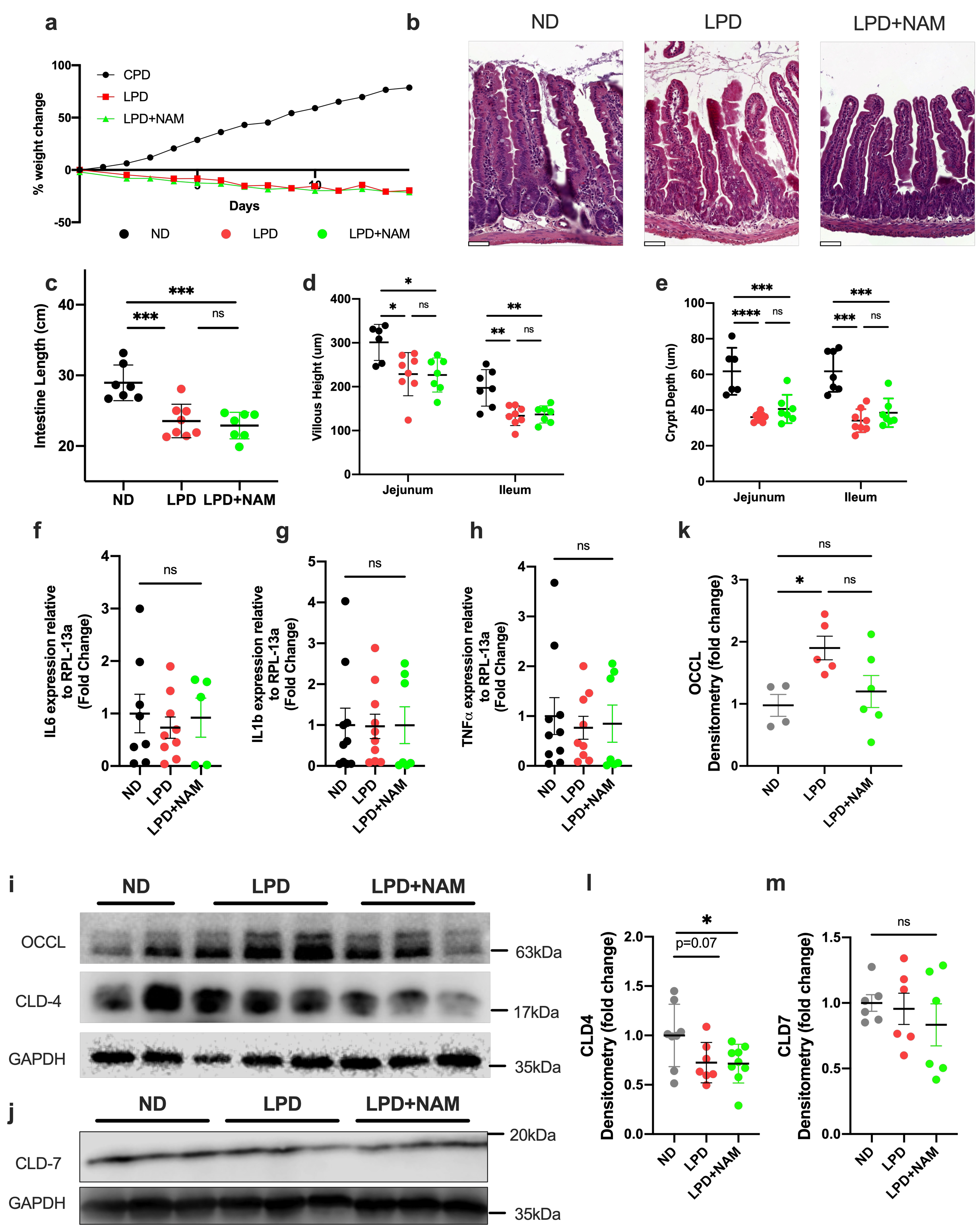
**

**Supplemental Figure 2. Morphometric alterations in response to nicotinamide supplementation.** (**a**) Body weight over the period of 14 days. Individual data points are shown with mean and error bars that indicate the SD, n= 6 mice per group (**b**) Representative H&E-stained images of jejunum from mice exposed to each diet, n=8 mice per group (scale bar = 50um) **(c)** The average average intestine length, **(d-e)** average measured villus height and crypt depth in the jejunum and ileum Bar graph indicate the mean with SD **(f-g)** Gene expression analysis for pro-inflammatory cytokines IL6, IL1b, TNFa. (Ordinary Anova with Tukey Post-hoc comparison). (**I,j**) Immunoblotting of OCCL, CLD-4, CLD-7, and loading control GAPDH. (**k-m**) Corresponding densitometry quantification of immunoblots. Bars indicate the mean with SD, n=5-6 per group (Ordinary ANOVA with Tukey’s post-hoc) *** P< 0.001, ** P<0.01, *p<0.05

**
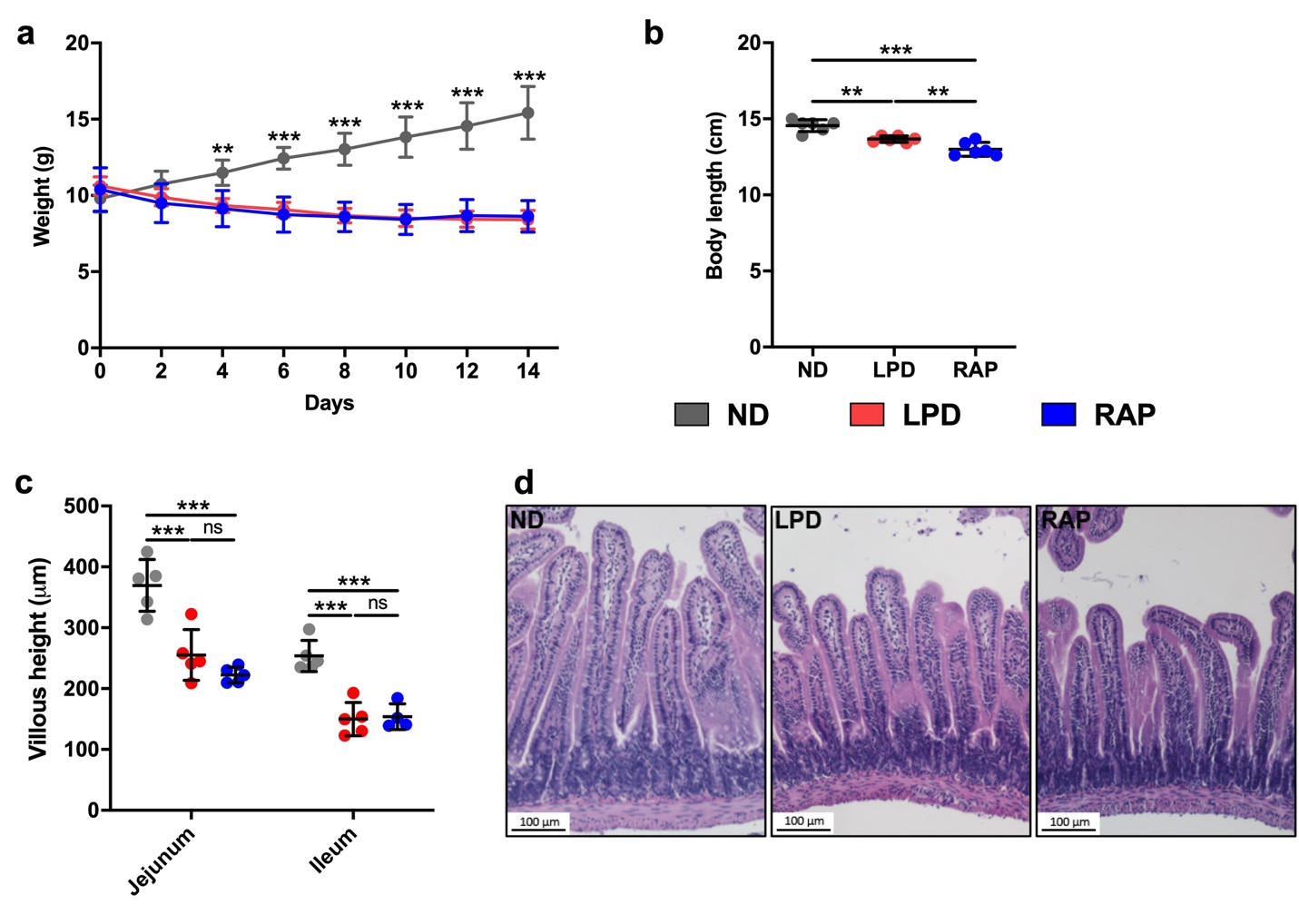
**

**Supplementary figure 3. Morphometric alterations in response to rapamycin treatment (a**) Body weight over the period of 14 days. Individual data points are shown with mean and error bars that indicate the SD, n=6 mice per group (** P<0.01, *** P<0.001, repeated measures analysis of variance). After 2 weeks, the total body length (**b**) was measured. Individual data points are shown with mean and error bars that indicate the SD, n=6 mice per group (* P<0.05, ** P<0.01, *** P<0.001, Two-way ANOVA with Tukey’s post-hoc analysis). (**c**) The average measured villus height in the jejunum and ileum with representative H&E-stained images of jejunum (**d**) from each experimental group. Scale bar, 100 µm. Bar graph indicated the mean with SD, n= 5 mice per group (*** P<0.001, Two-way ANOVA with Tukey’s post-hoc analysis)


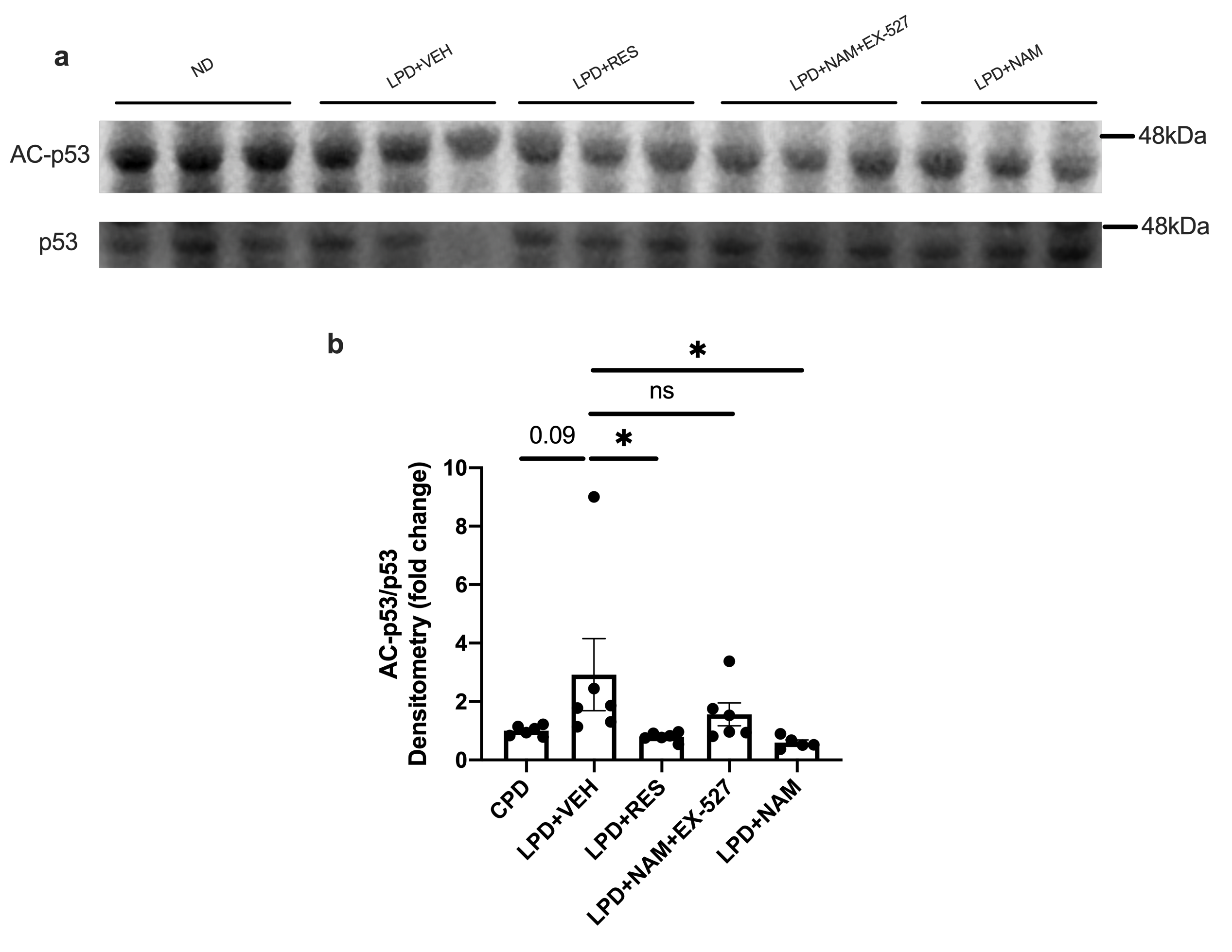


**Supplementary figure 4. SIRT1 deacetylase activity altered in response to SIRT1 activation or inhibition (a**) Immunoblotting of AC-P53 and P53. (**b**) Corresponding densitometry quantification of immunoblots. Bars indicate the mean with SEM, n=5-6 per group (Ordinary ANOVA with Tukey’s post-hoc) *p<0.05
