# Supplementary material for: Rebalancing of mitochondrial homeostasis through an NAD+-SIRT1 pathway preserves intestinal barrier function in severe malnutrition
Source: eBioMedicine. 2023 Sep 20;96:104809. doi: 10.1016/j.ebiom.2023.104809 (PMC10520344; doi:10.1016/j.ebiom.2023.104809)
Supplement: Western Blot Full gels [file mmc2.pptx]

## Slide 1
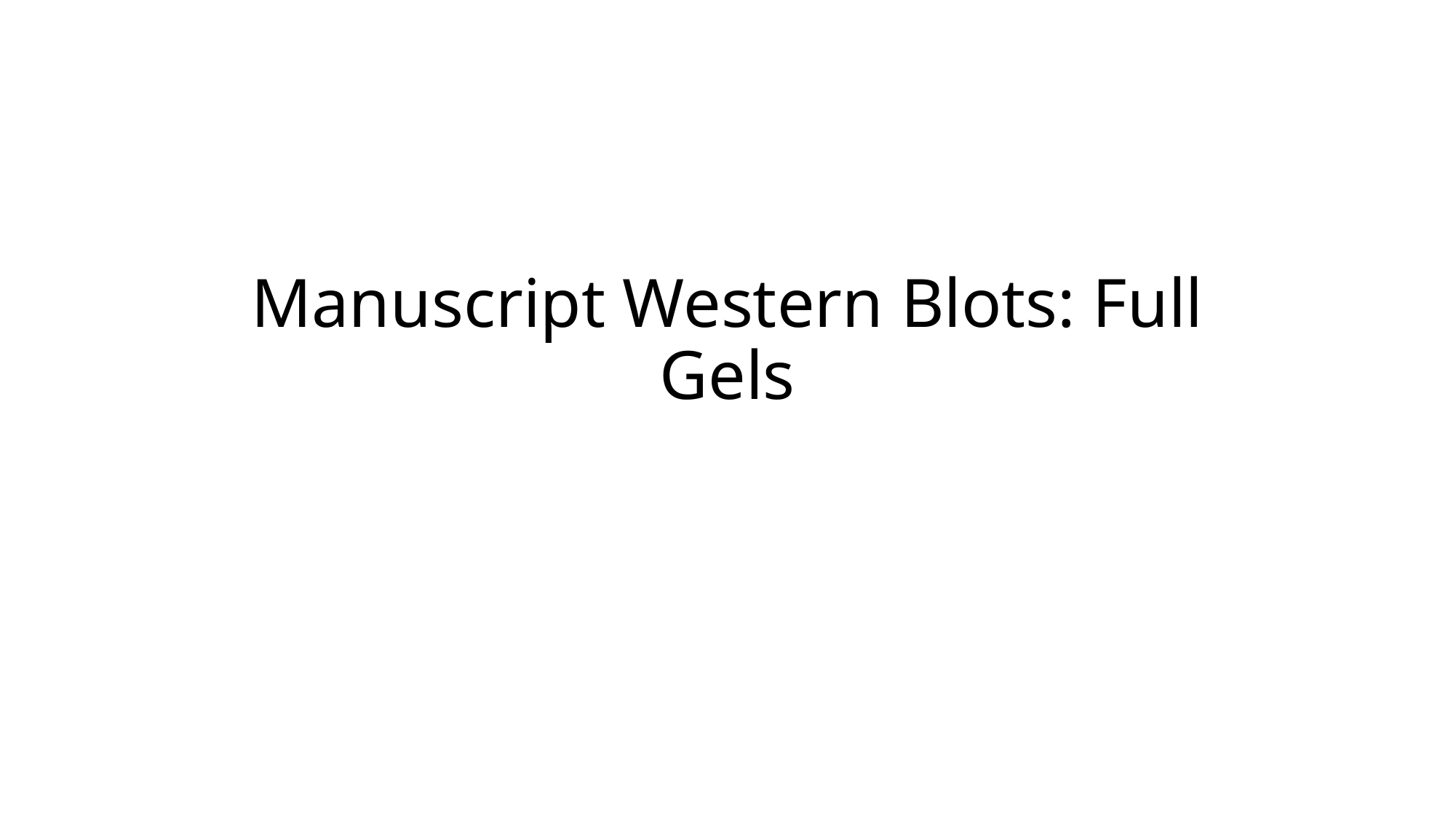

# Manuscript Western Blots: Full Gels

## Slide 2
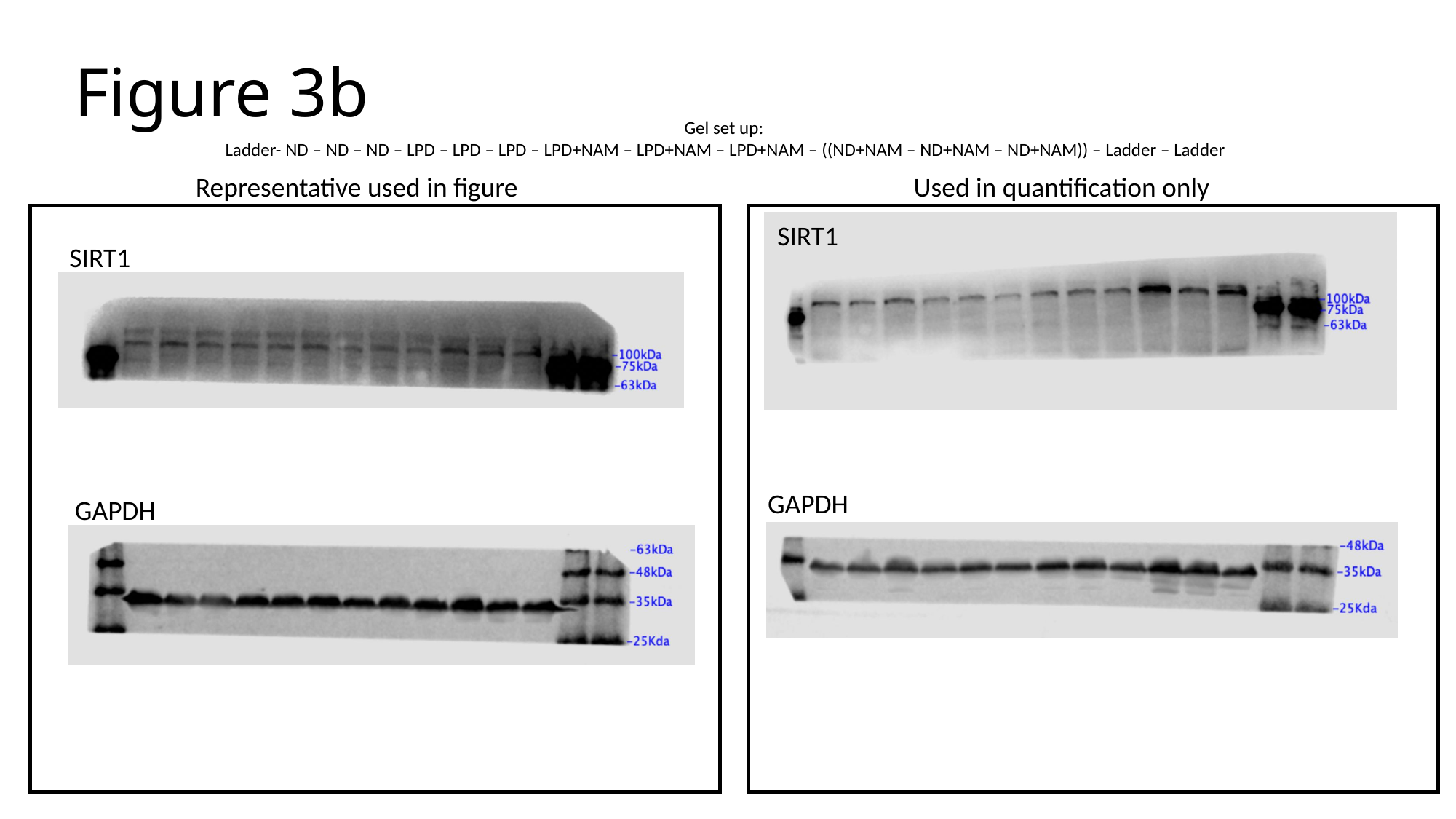

# Figure 3b
Gel set up:
Ladder- ND – ND – ND – LPD – LPD – LPD – LPD+NAM – LPD+NAM – LPD+NAM – ((ND+NAM – ND+NAM – ND+NAM)) – Ladder – Ladder
Representative used in figure
Used in quantification only
SIRT1
SIRT1
GAPDH
GAPDH

## Slide 3
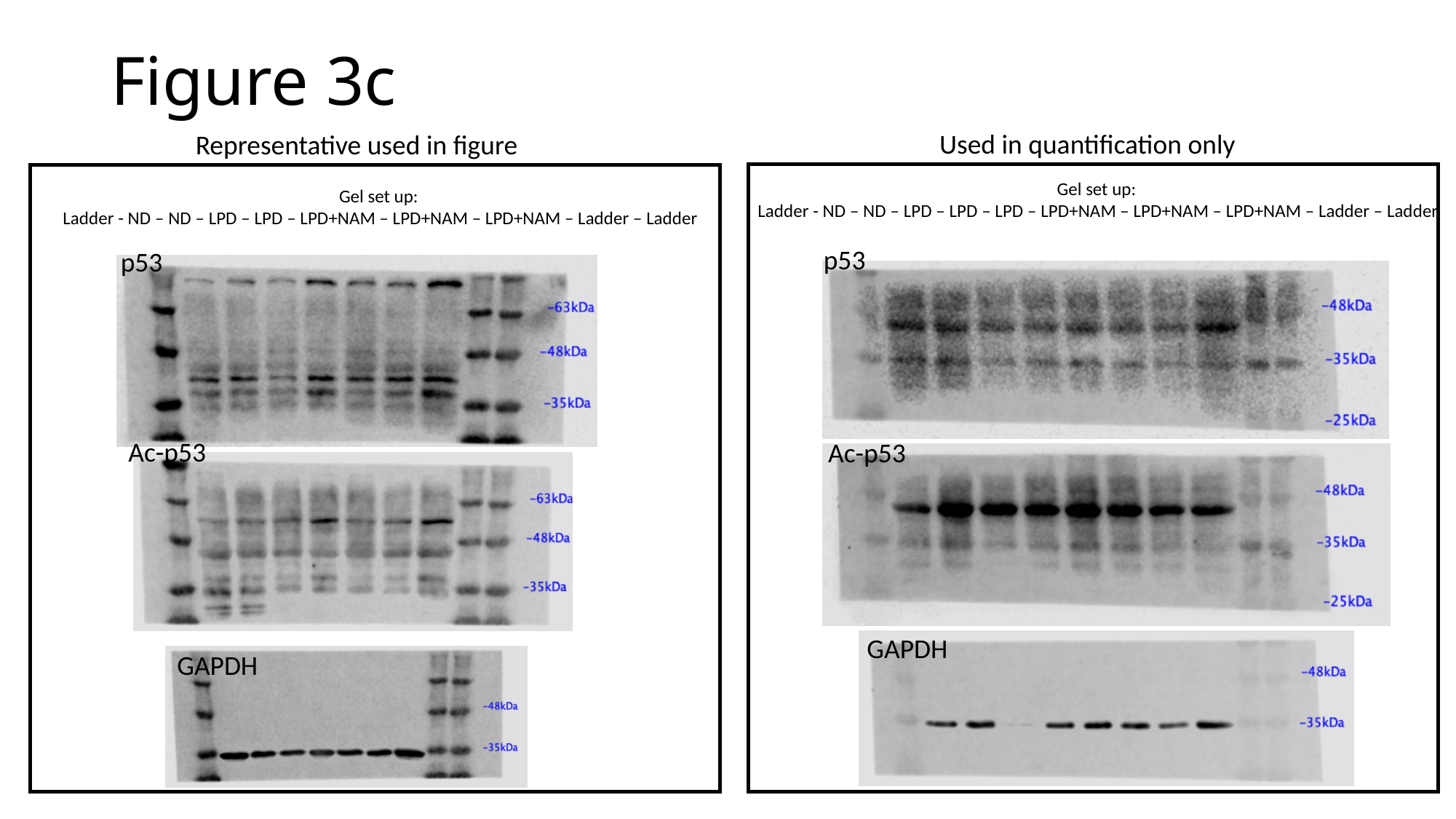

# Figure 3c
Used in quantification only
Representative used in figure
Gel set up:
Ladder - ND – ND – LPD – LPD – LPD – LPD+NAM – LPD+NAM – LPD+NAM – Ladder – Ladder
Gel set up:
Ladder - ND – ND – LPD – LPD – LPD+NAM – LPD+NAM – LPD+NAM – Ladder – Ladder
p53
p53
Ac-p53
Ac-p53
GAPDH
GAPDH

## Slide 4
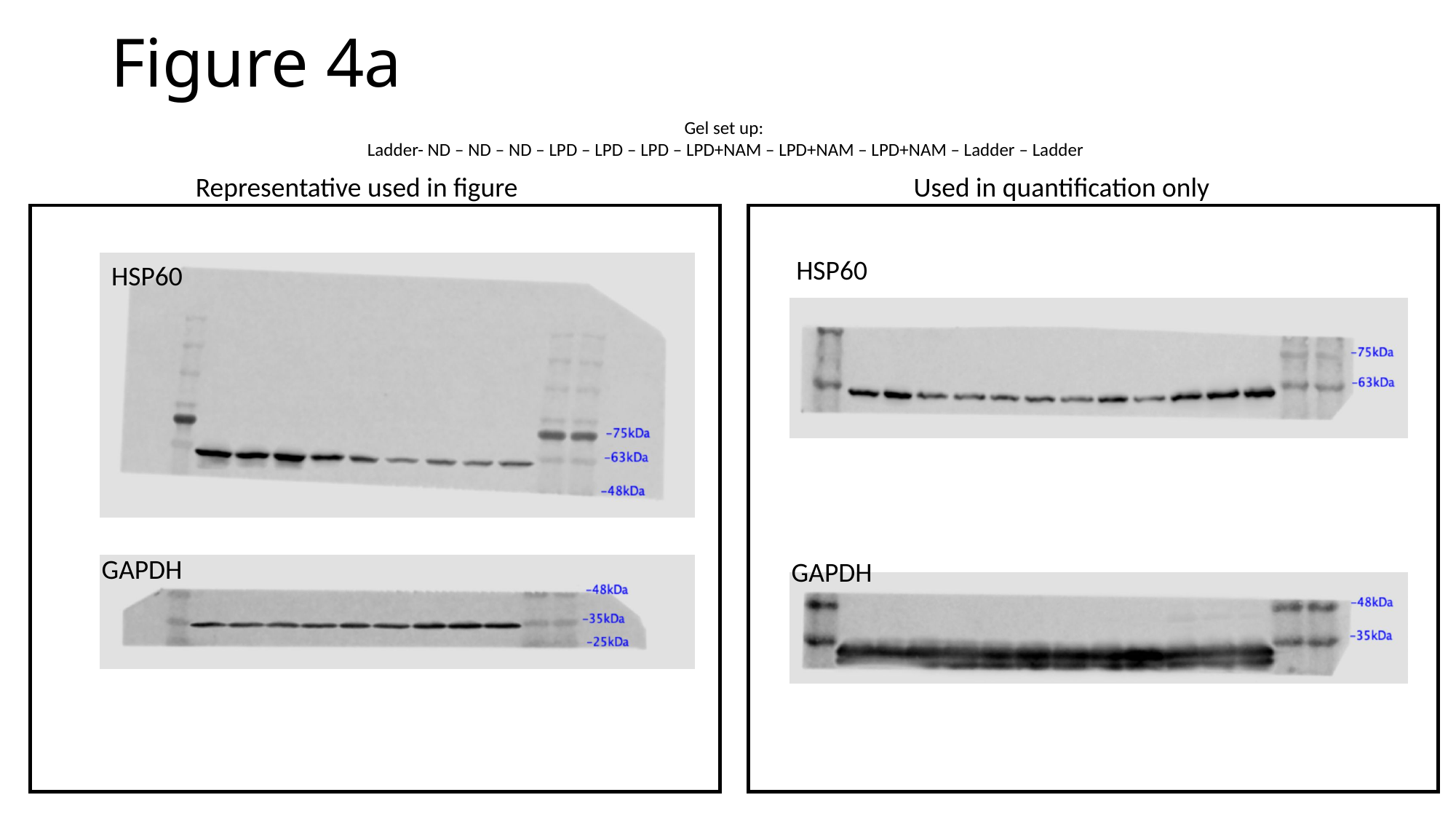

# Figure 4a
Gel set up:
Ladder- ND – ND – ND – LPD – LPD – LPD – LPD+NAM – LPD+NAM – LPD+NAM – Ladder – Ladder
Representative used in figure
Used in quantification only
HSP60
HSP60
GAPDH
GAPDH

## Slide 5
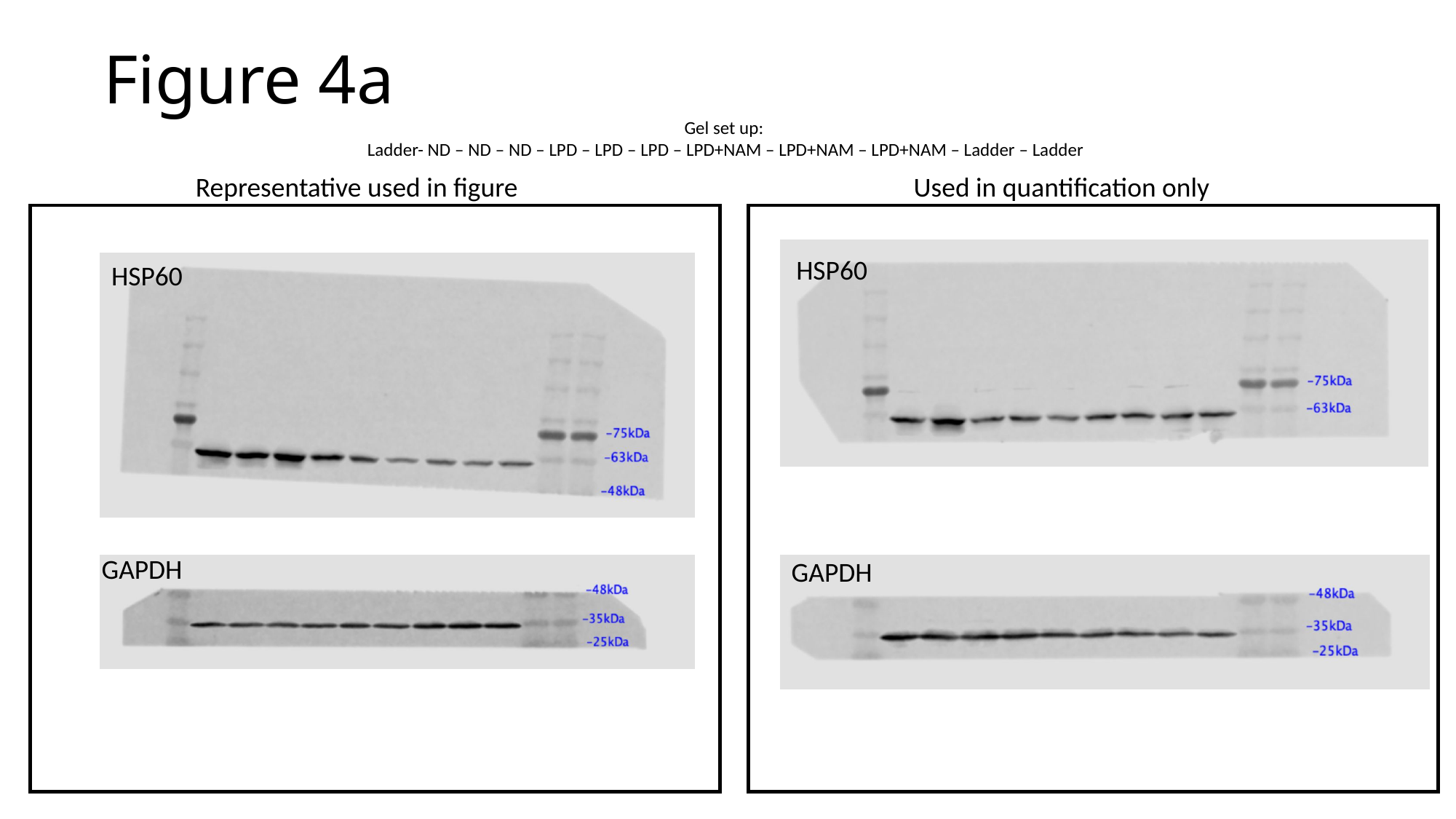

# Figure 4a
Gel set up:
Ladder- ND – ND – ND – LPD – LPD – LPD – LPD+NAM – LPD+NAM – LPD+NAM – Ladder – Ladder
Representative used in figure
Used in quantification only
HSP60
HSP60
GAPDH
GAPDH

## Slide 6
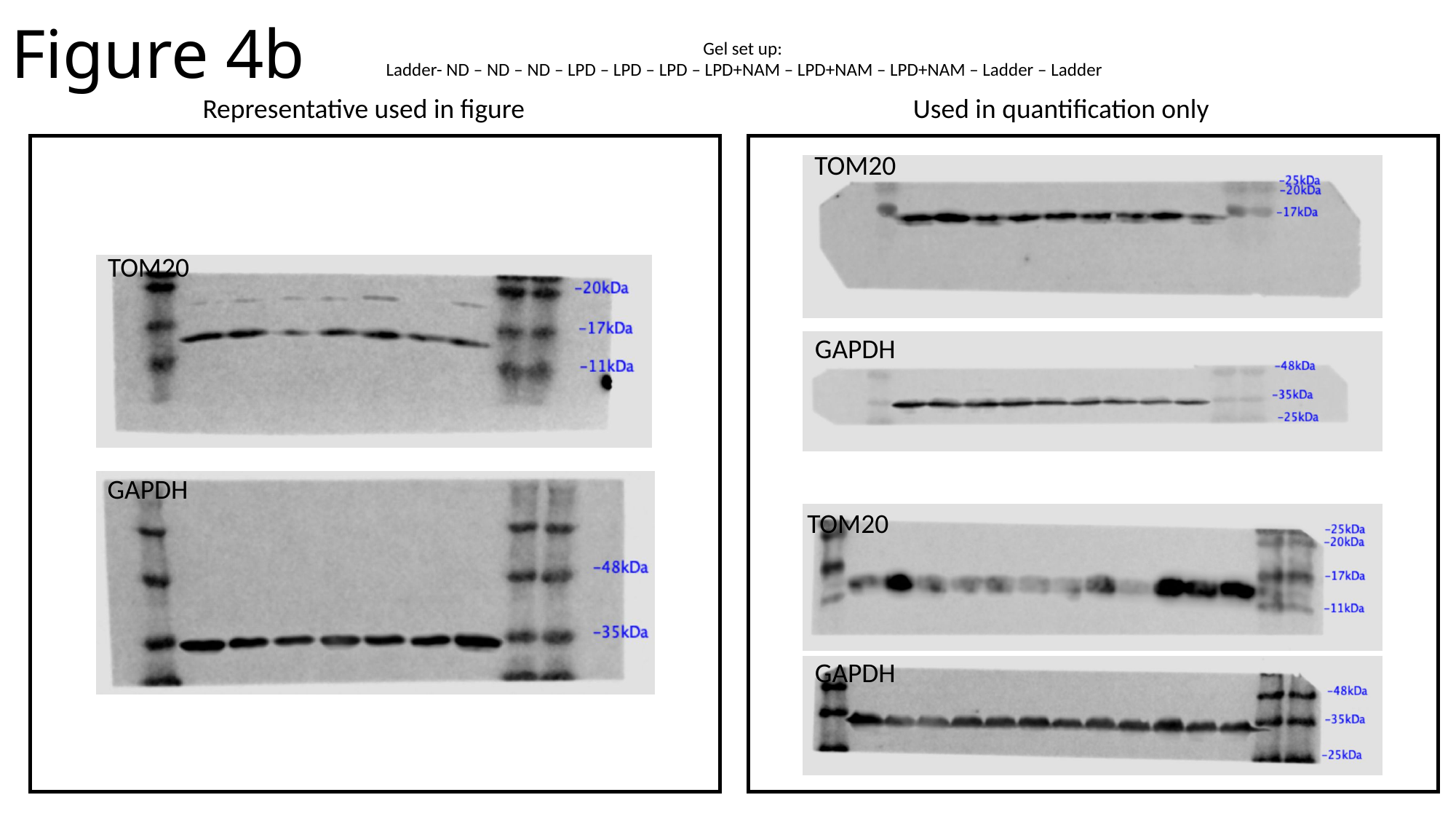

# Figure 4b
Gel set up:
Ladder- ND – ND – ND – LPD – LPD – LPD – LPD+NAM – LPD+NAM – LPD+NAM – Ladder – Ladder
Representative used in figure
Used in quantification only
TOM20
TOM20
GAPDH
GAPDH
TOM20
GAPDH

## Slide 7
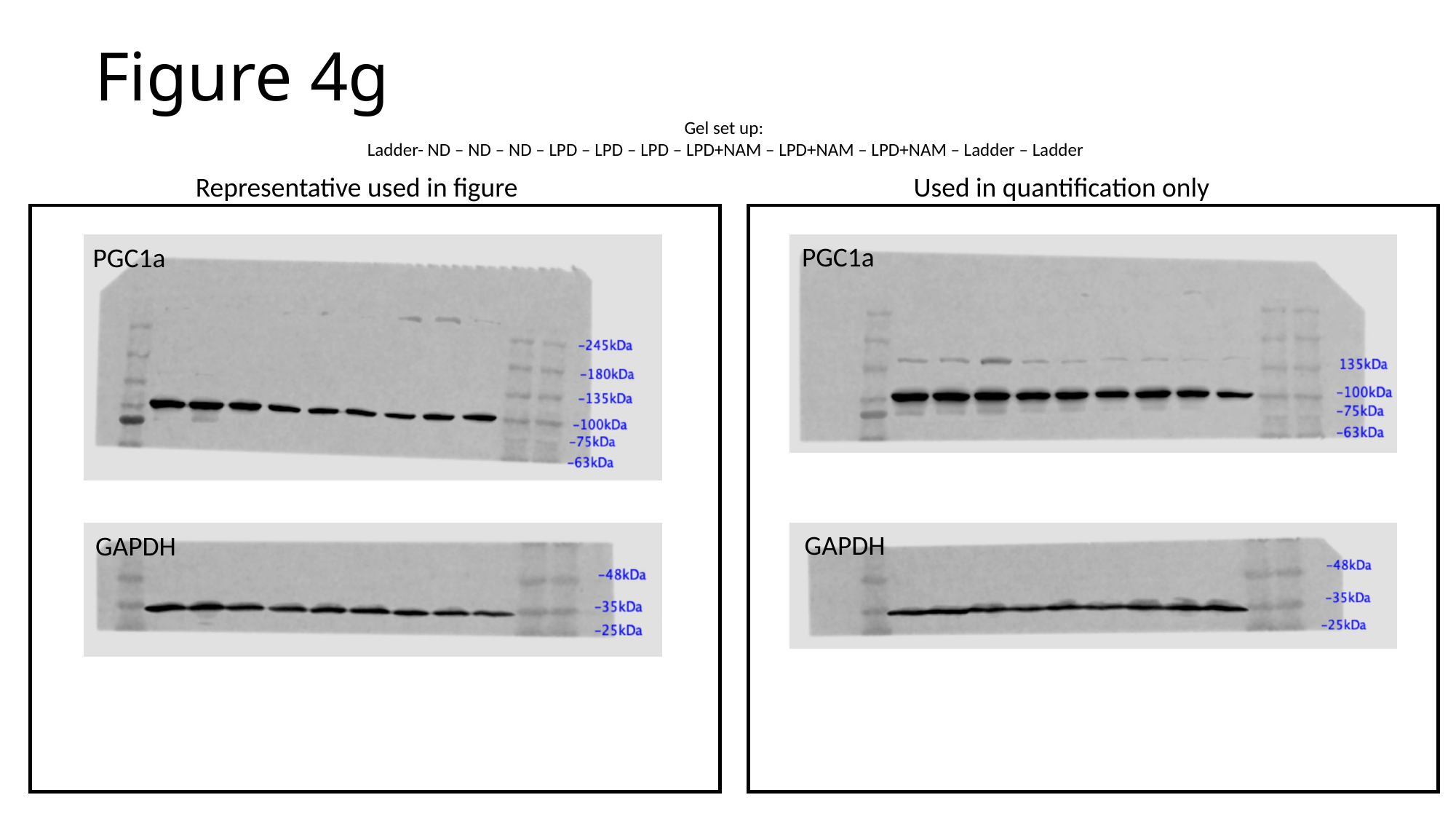

# Figure 4g
Gel set up:
Ladder- ND – ND – ND – LPD – LPD – LPD – LPD+NAM – LPD+NAM – LPD+NAM – Ladder – Ladder
Representative used in figure
Used in quantification only
PGC1a
PGC1a
GAPDH
GAPDH

## Slide 8
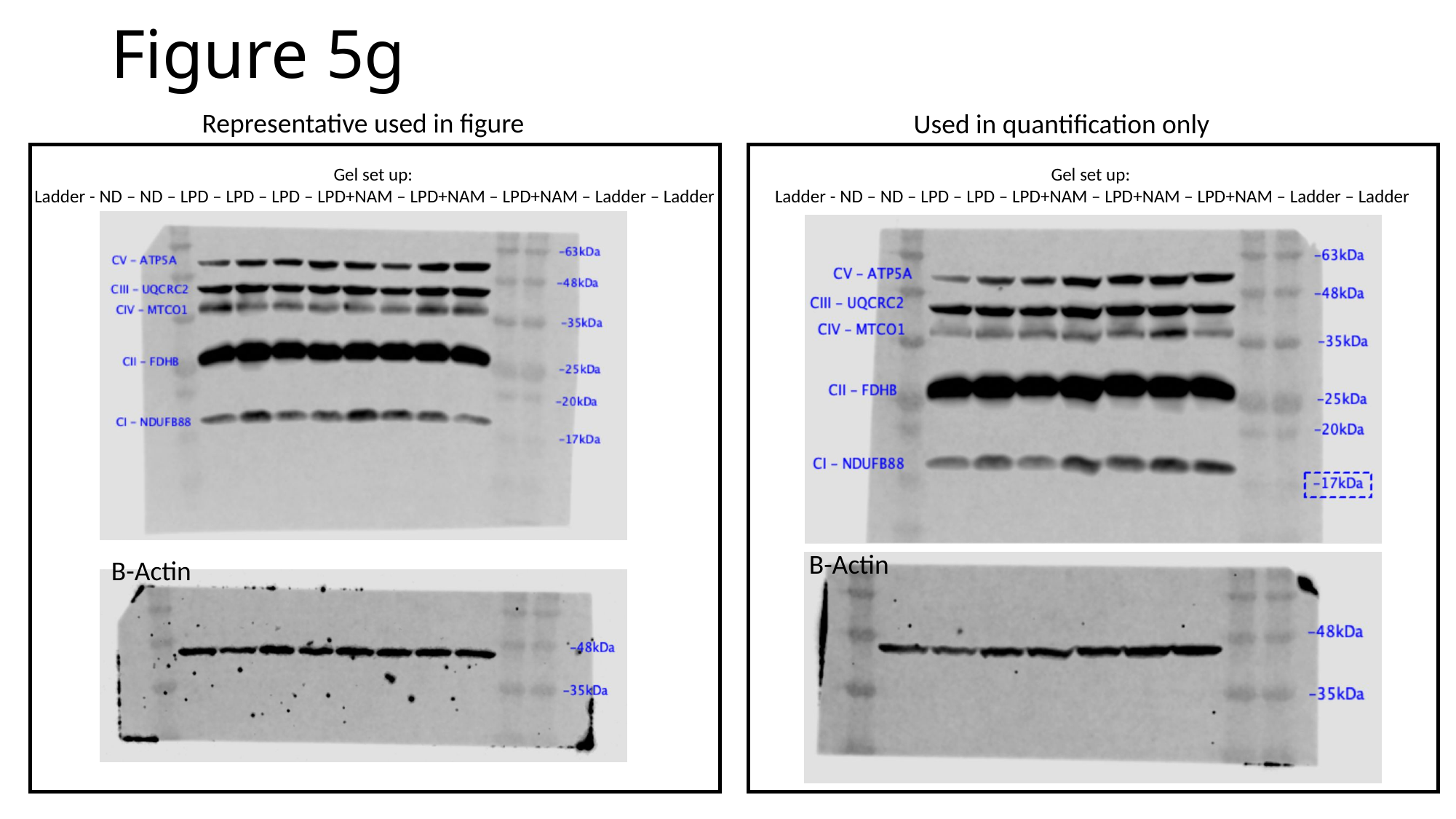

# Figure 5g
Representative used in figure
Used in quantification only
Gel set up:
Ladder - ND – ND – LPD – LPD – LPD+NAM – LPD+NAM – LPD+NAM – Ladder – Ladder
Gel set up:
Ladder - ND – ND – LPD – LPD – LPD – LPD+NAM – LPD+NAM – LPD+NAM – Ladder – Ladder
B-Actin
B-Actin

## Slide 9
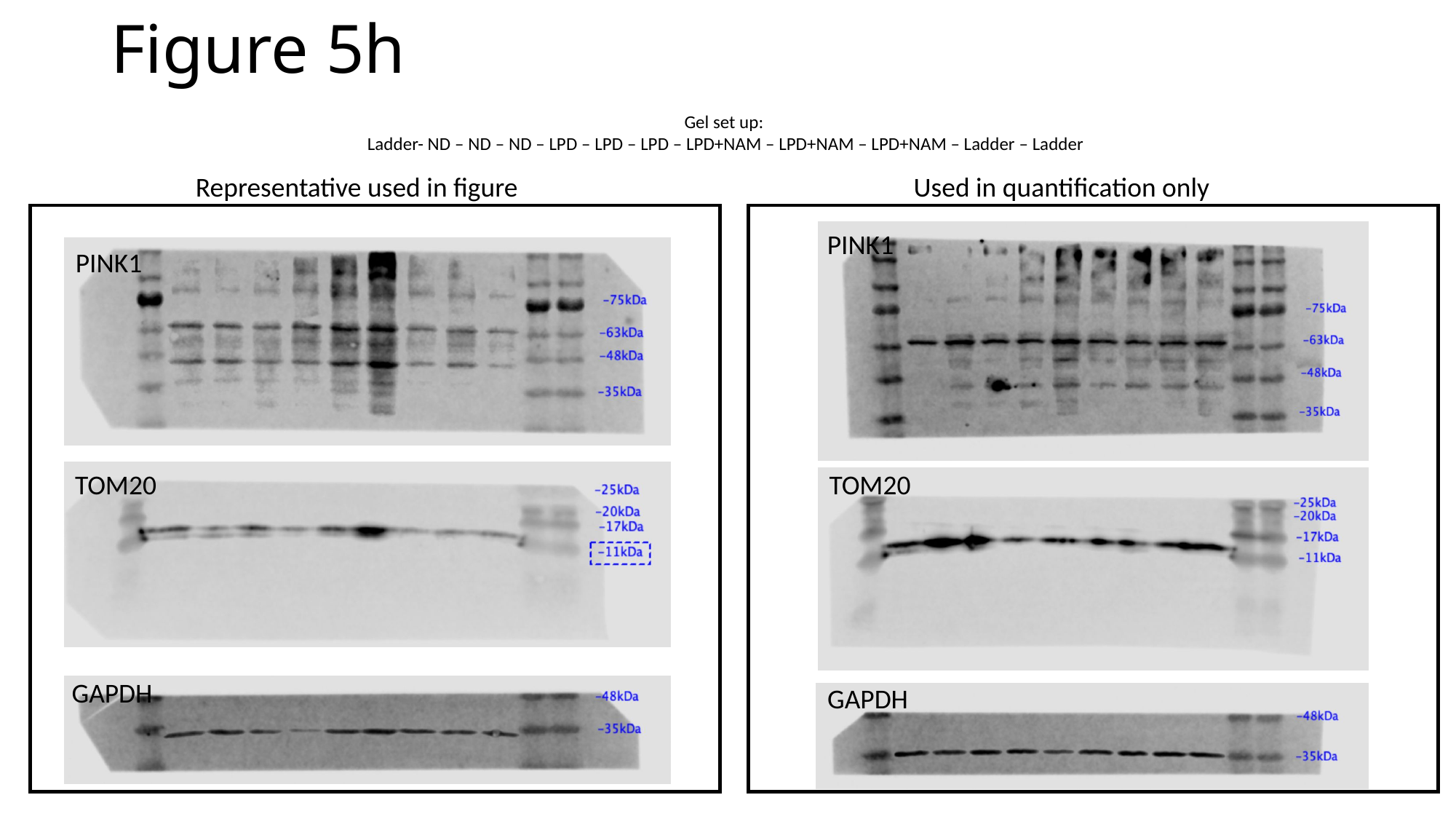

# Figure 5h
Gel set up:
Ladder- ND – ND – ND – LPD – LPD – LPD – LPD+NAM – LPD+NAM – LPD+NAM – Ladder – Ladder
Representative used in figure
Used in quantification only
PINK1
PINK1
TOM20
TOM20
GAPDH
GAPDH

## Slide 10
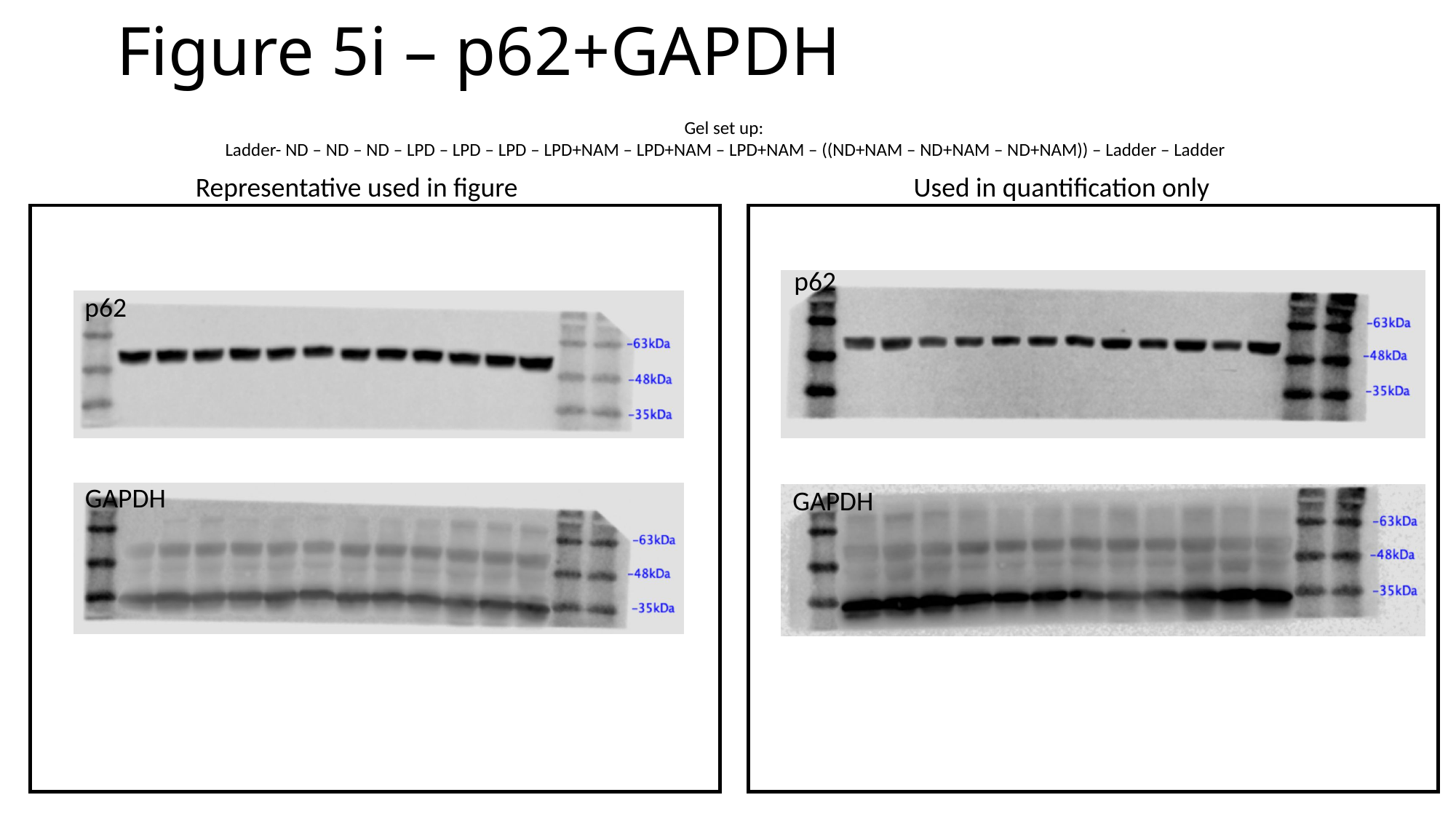

# Figure 5i – p62+GAPDH
Gel set up:
Ladder- ND – ND – ND – LPD – LPD – LPD – LPD+NAM – LPD+NAM – LPD+NAM – ((ND+NAM – ND+NAM – ND+NAM)) – Ladder – Ladder
Representative used in figure
Used in quantification only
p62
p62
GAPDH
GAPDH

## Slide 11
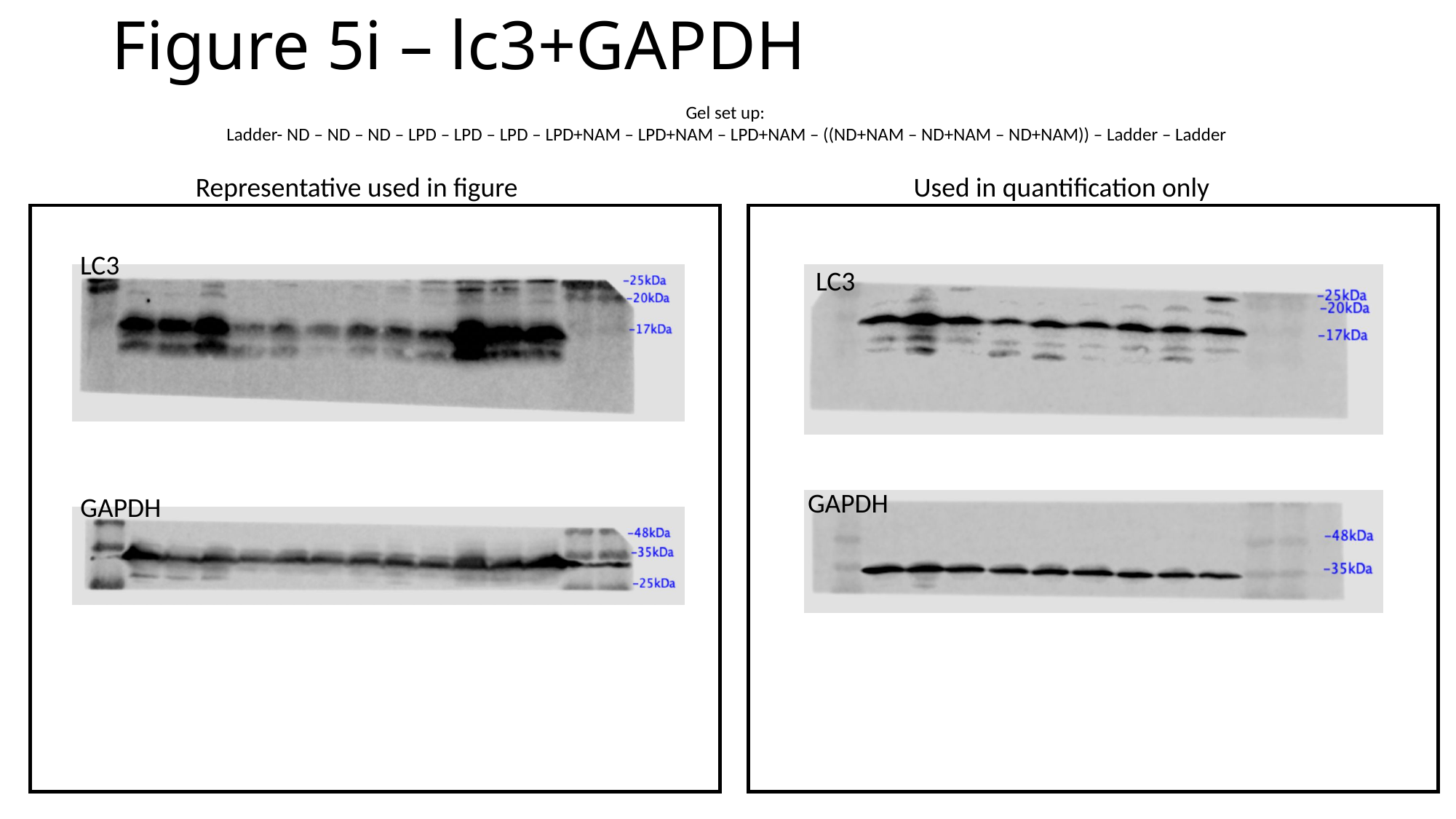

# Figure 5i – lc3+GAPDH
Gel set up:
Ladder- ND – ND – ND – LPD – LPD – LPD – LPD+NAM – LPD+NAM – LPD+NAM – ((ND+NAM – ND+NAM – ND+NAM)) – Ladder – Ladder
Representative used in figure
Used in quantification only
LC3
LC3
GAPDH
GAPDH

## Slide 12
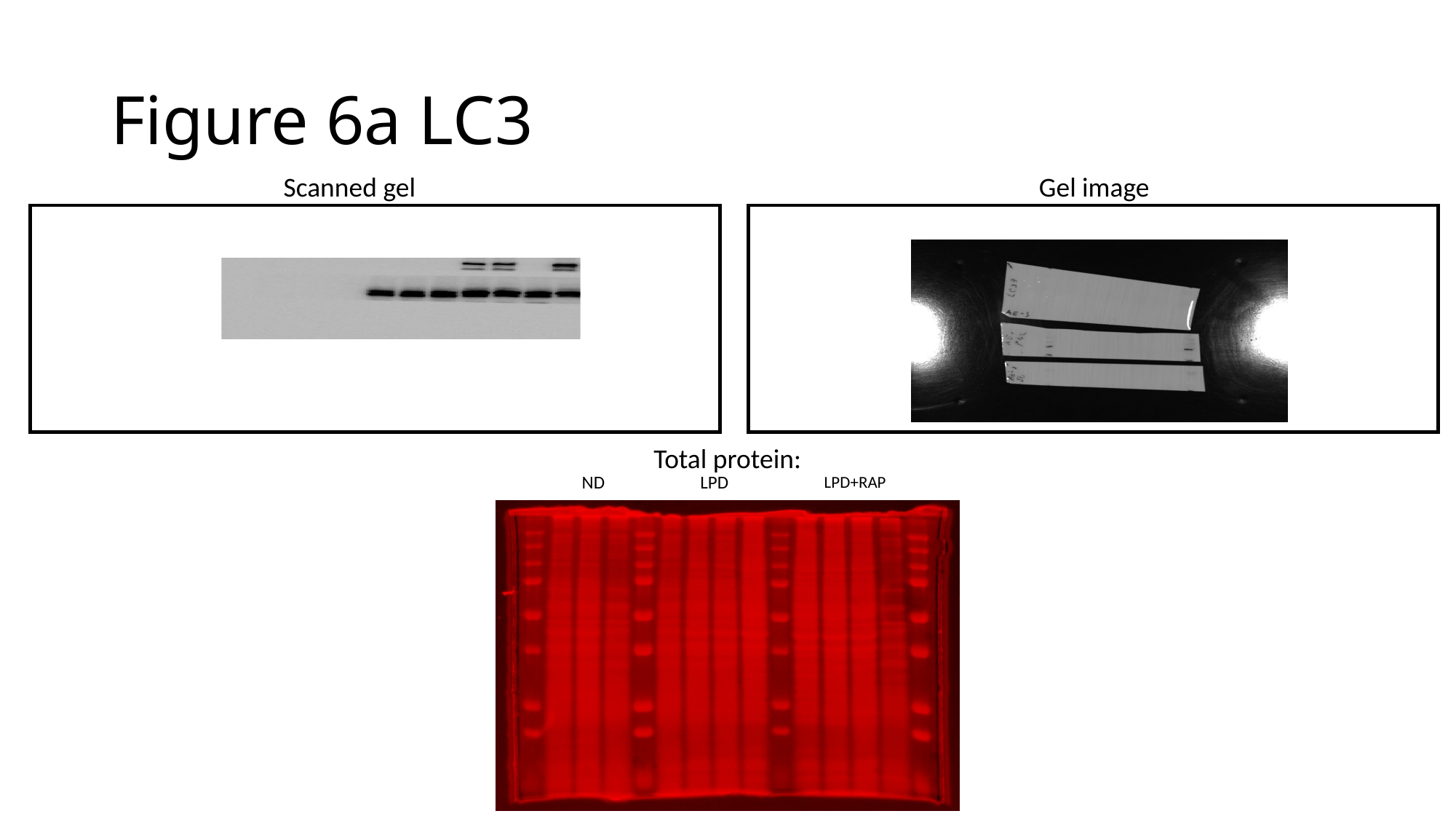

# Figure 6a LC3
Scanned gel
Gel image
Total protein:
ND
LPD
LPD+RAP

## Slide 13
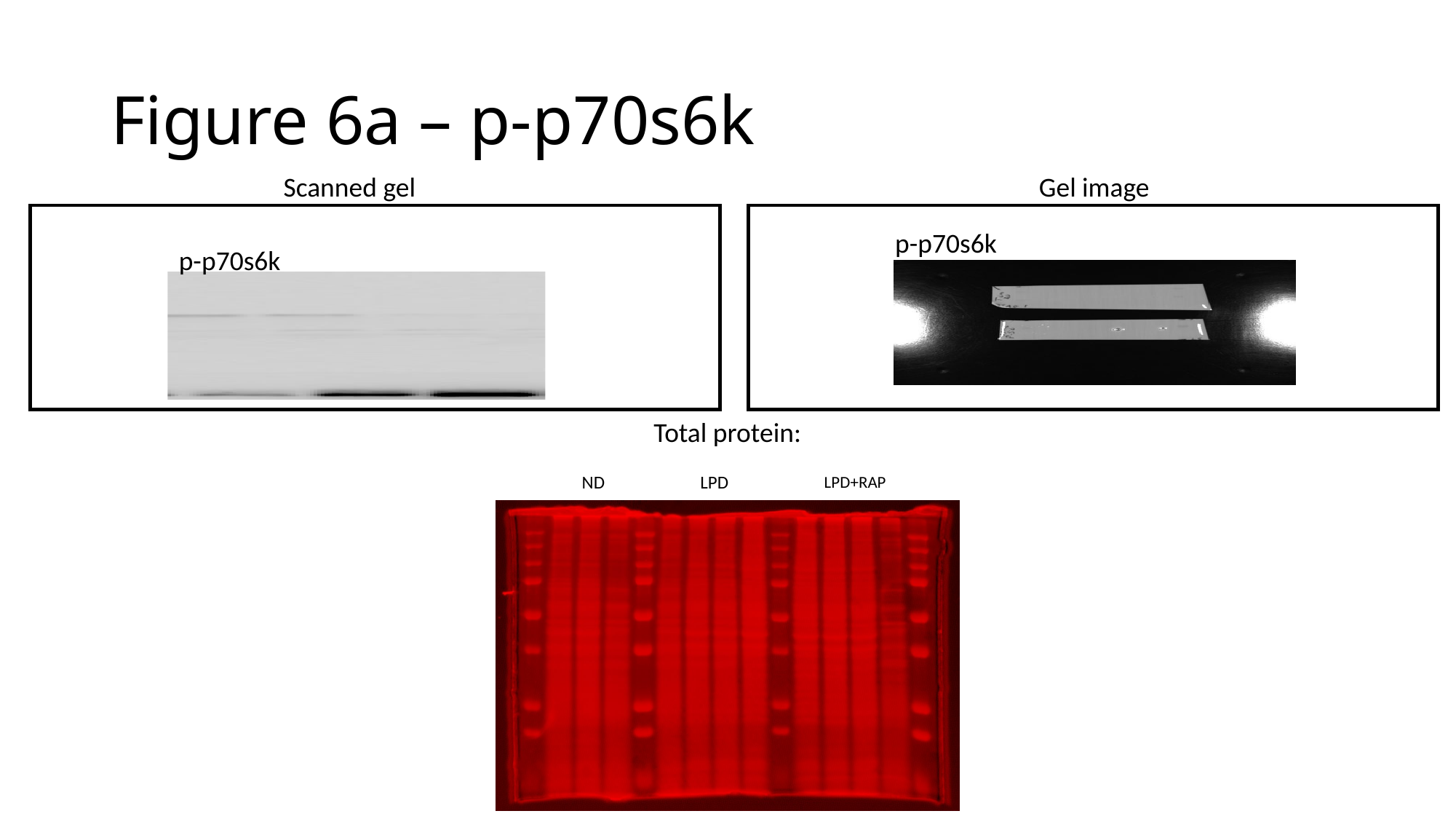

# Figure 6a – p-p70s6k
Scanned gel
Gel image
p-p70s6k
p-p70s6k
Total protein:
ND
LPD
LPD+RAP

## Slide 14
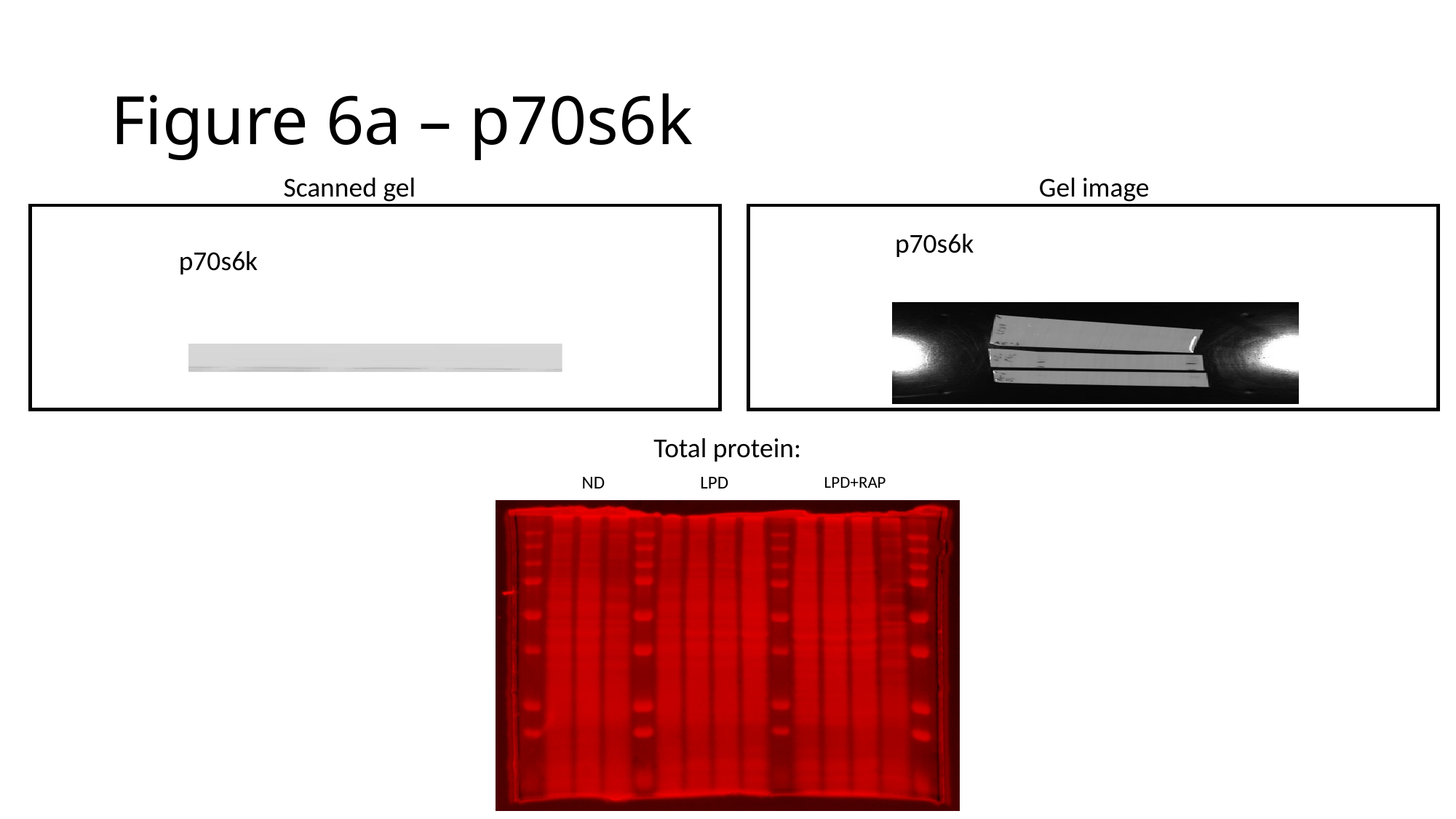

# Figure 6a – p70s6k
Scanned gel
Gel image
p70s6k
p70s6k
Total protein:
ND
LPD
LPD+RAP

## Slide 15
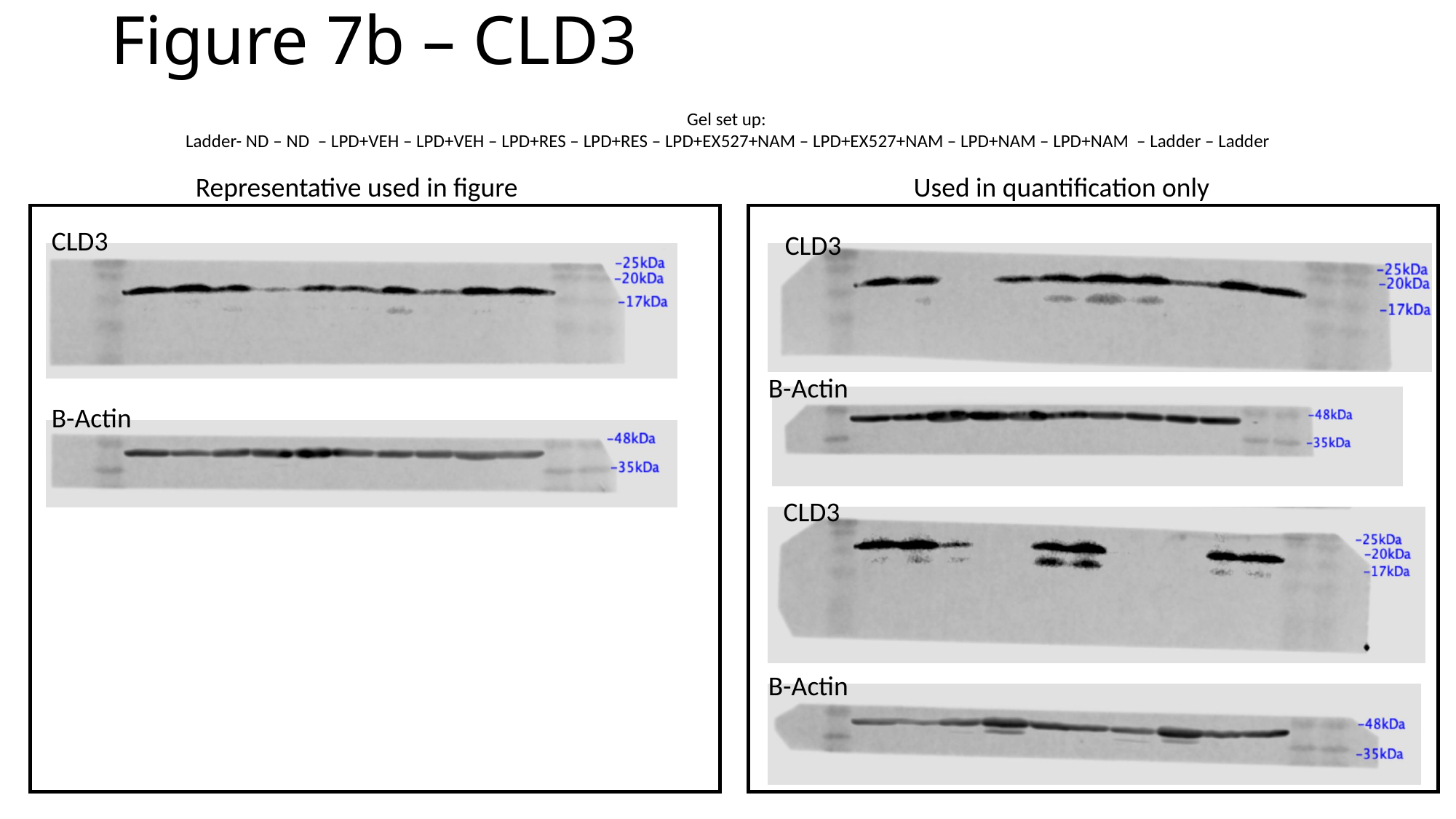

# Figure 7b – CLD3
Gel set up:
Ladder- ND – ND – LPD+VEH – LPD+VEH – LPD+RES – LPD+RES – LPD+EX527+NAM – LPD+EX527+NAM – LPD+NAM – LPD+NAM – Ladder – Ladder
Representative used in figure
Used in quantification only
CLD3
CLD3
B-Actin
B-Actin
CLD3
B-Actin

## Slide 16
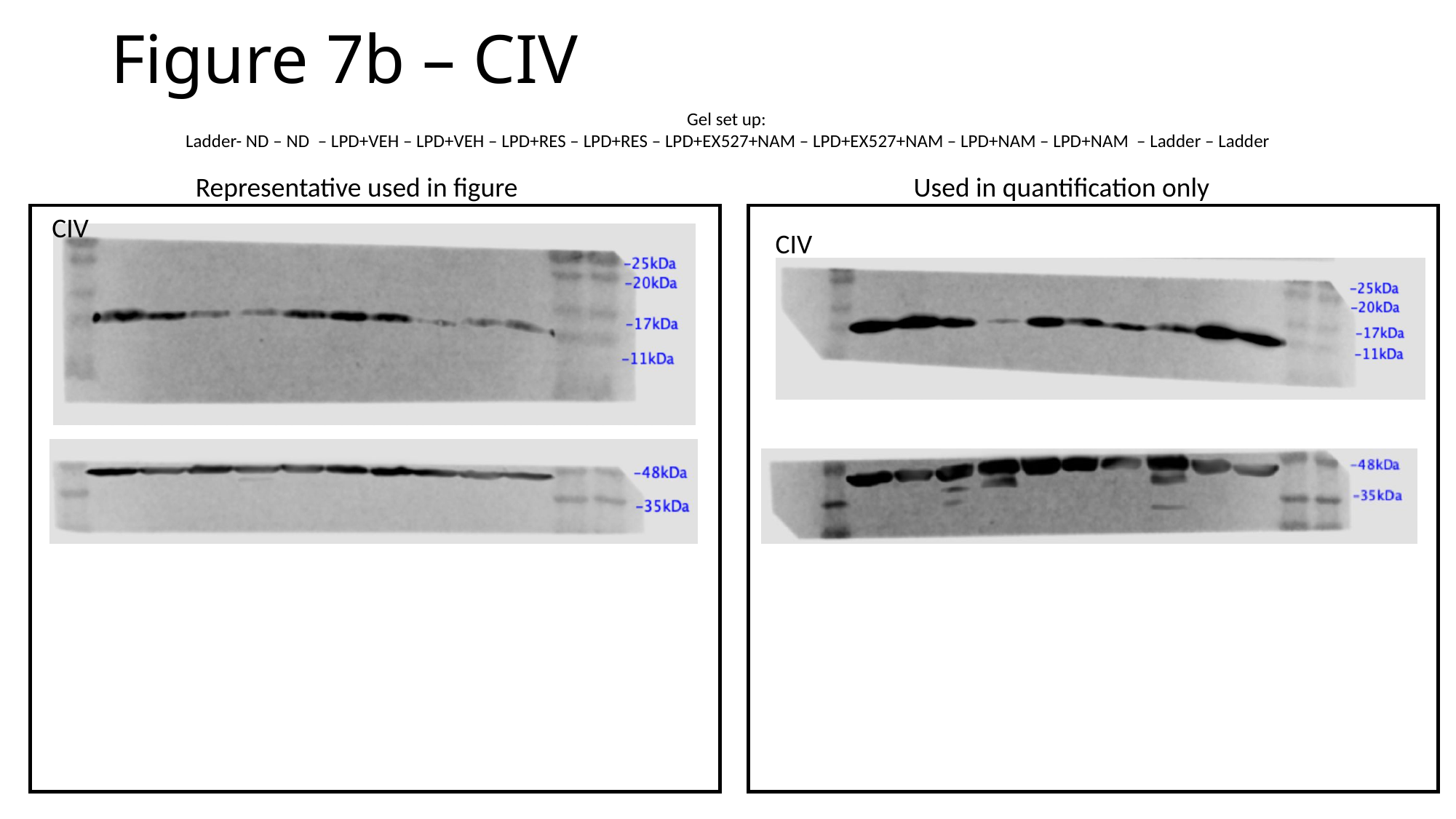

# Figure 7b – CIV
Gel set up:
Ladder- ND – ND – LPD+VEH – LPD+VEH – LPD+RES – LPD+RES – LPD+EX527+NAM – LPD+EX527+NAM – LPD+NAM – LPD+NAM – Ladder – Ladder
Representative used in figure
Used in quantification only
CIV
CIV

## Slide 17
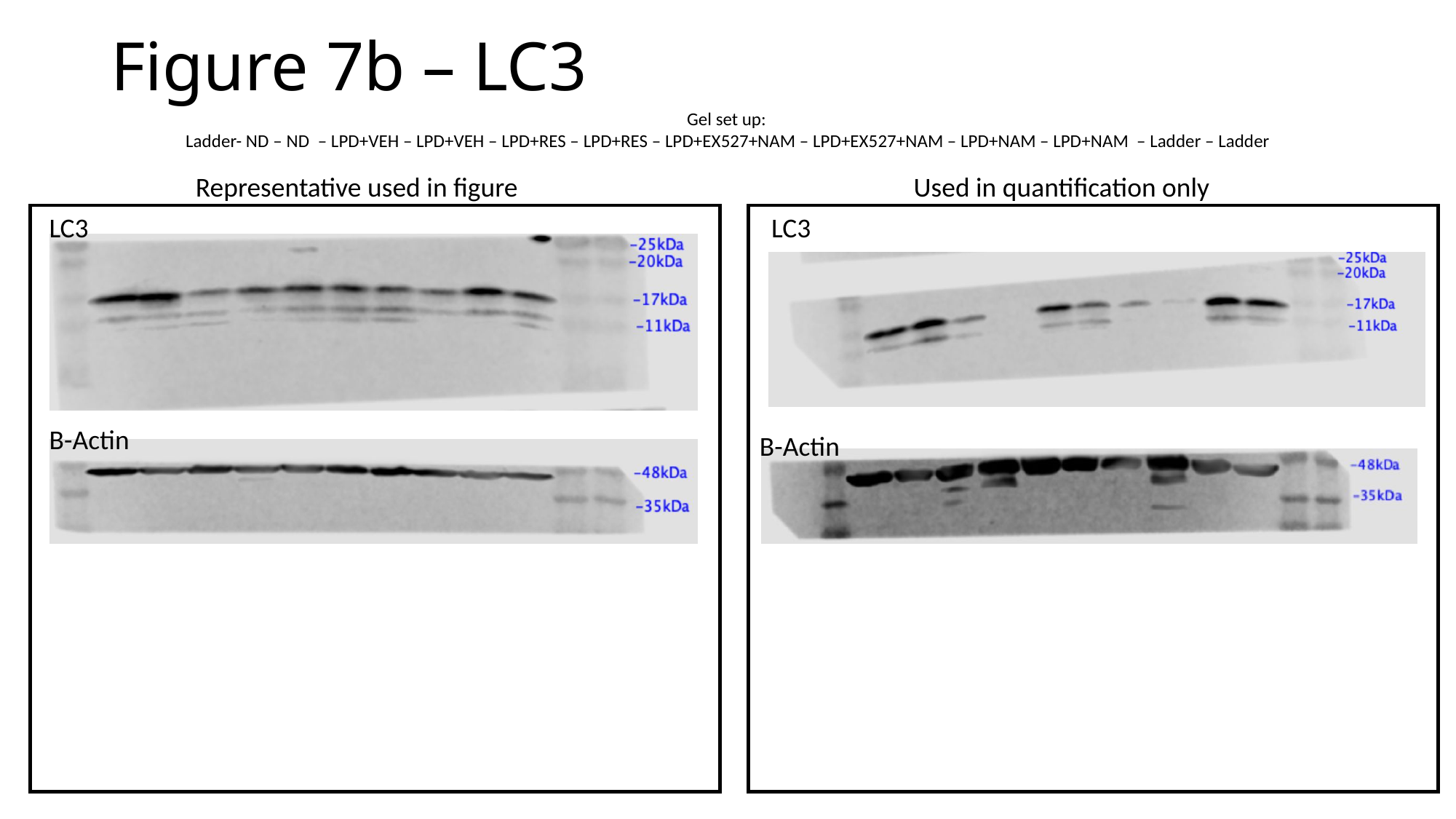

# Figure 7b – LC3
Gel set up:
Ladder- ND – ND – LPD+VEH – LPD+VEH – LPD+RES – LPD+RES – LPD+EX527+NAM – LPD+EX527+NAM – LPD+NAM – LPD+NAM – Ladder – Ladder
Representative used in figure
Used in quantification only
LC3
LC3
B-Actin
B-Actin

## Slide 18
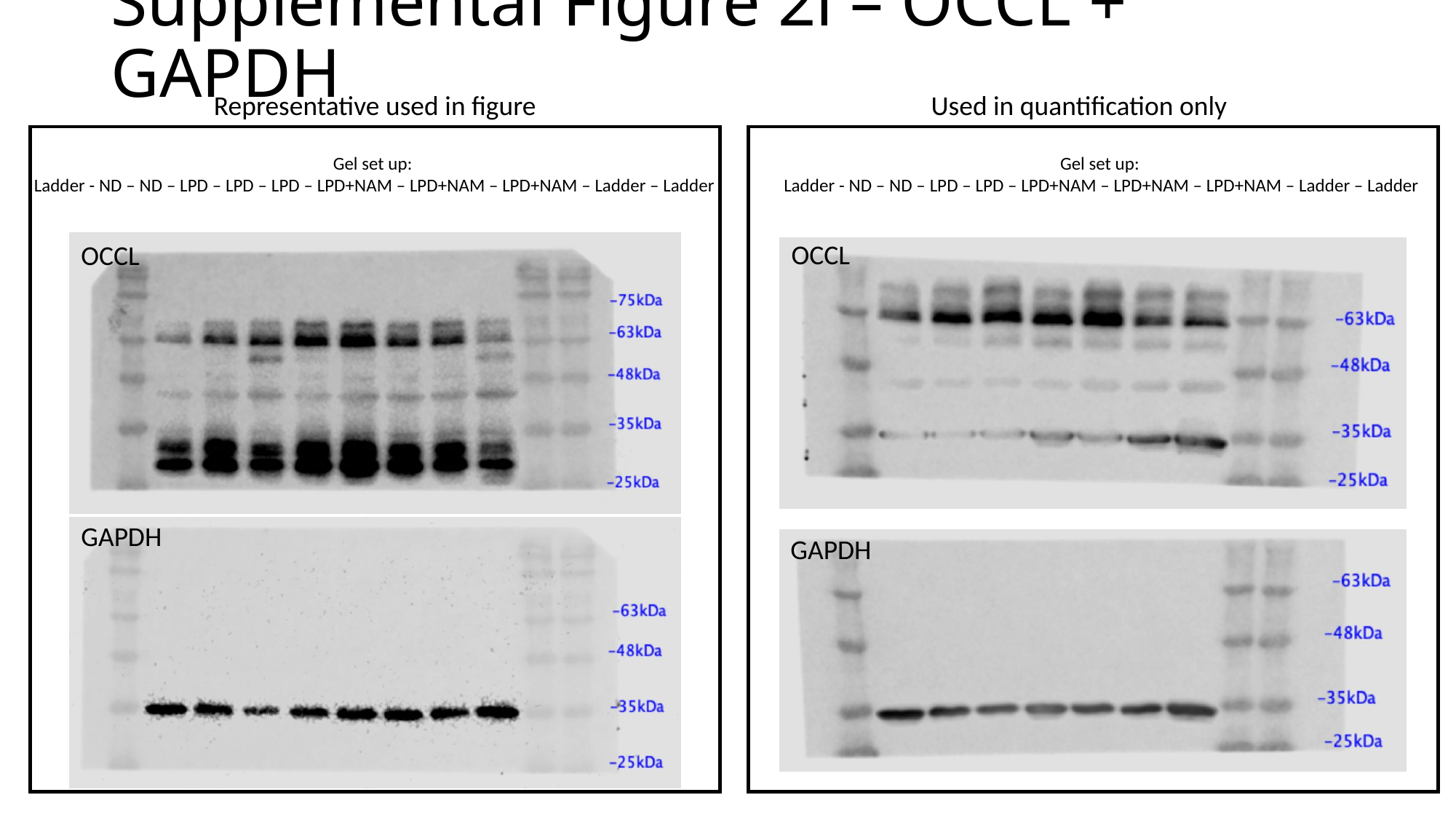

# Supplemental Figure 2i – OCCL + GAPDH
Representative used in figure
Used in quantification only
Gel set up:
Ladder - ND – ND – LPD – LPD – LPD – LPD+NAM – LPD+NAM – LPD+NAM – Ladder – Ladder
Gel set up:
Ladder - ND – ND – LPD – LPD – LPD+NAM – LPD+NAM – LPD+NAM – Ladder – Ladder
OCCL
OCCL
GAPDH
GAPDH

## Slide 19
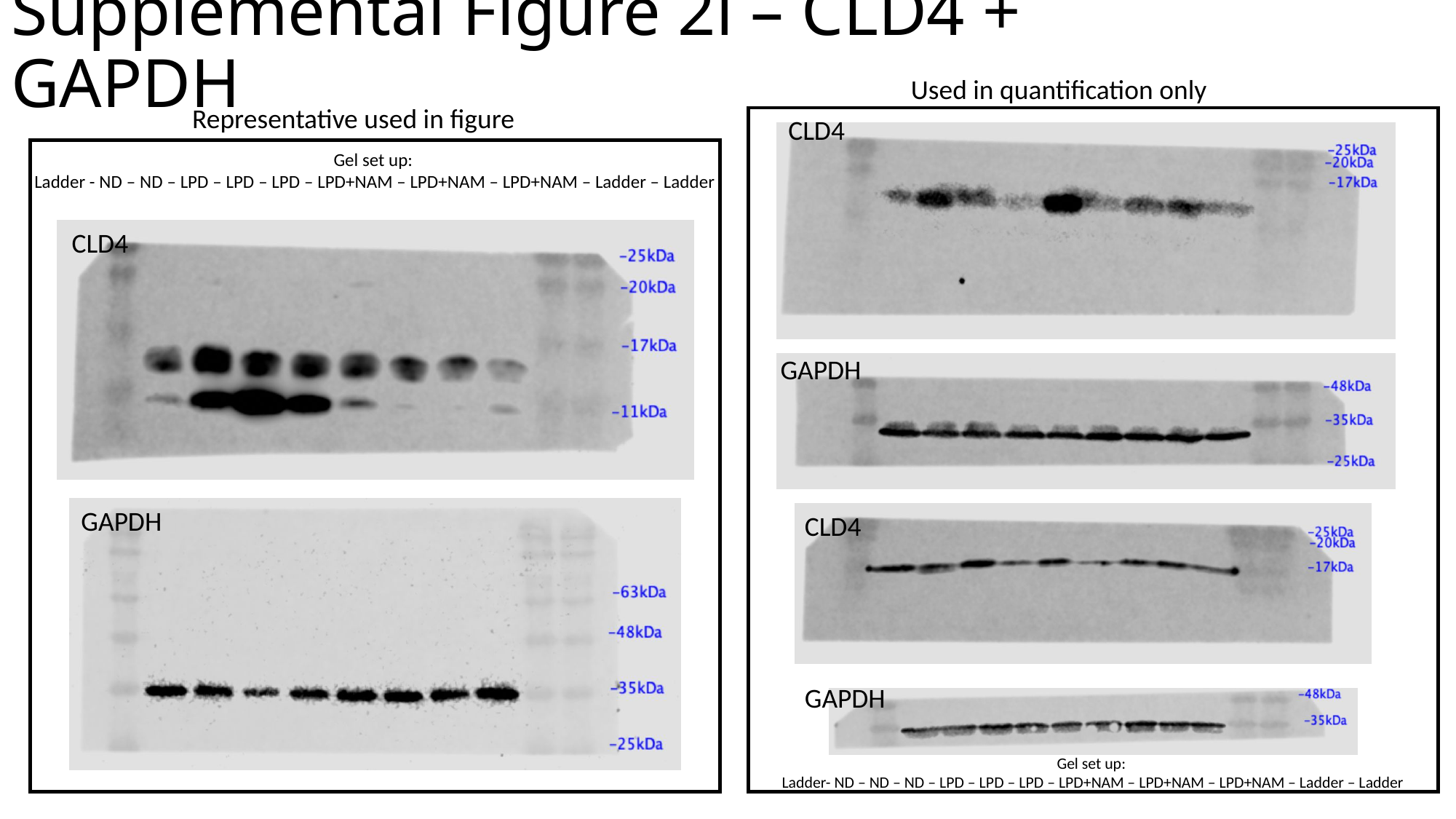

# Supplemental Figure 2i – CLD4 + GAPDH
Used in quantification only
Representative used in figure
CLD4
Gel set up:
Ladder - ND – ND – LPD – LPD – LPD – LPD+NAM – LPD+NAM – LPD+NAM – Ladder – Ladder
CLD4
GAPDH
GAPDH
CLD4
GAPDH
Gel set up:
Ladder- ND – ND – ND – LPD – LPD – LPD – LPD+NAM – LPD+NAM – LPD+NAM – Ladder – Ladder

## Slide 20
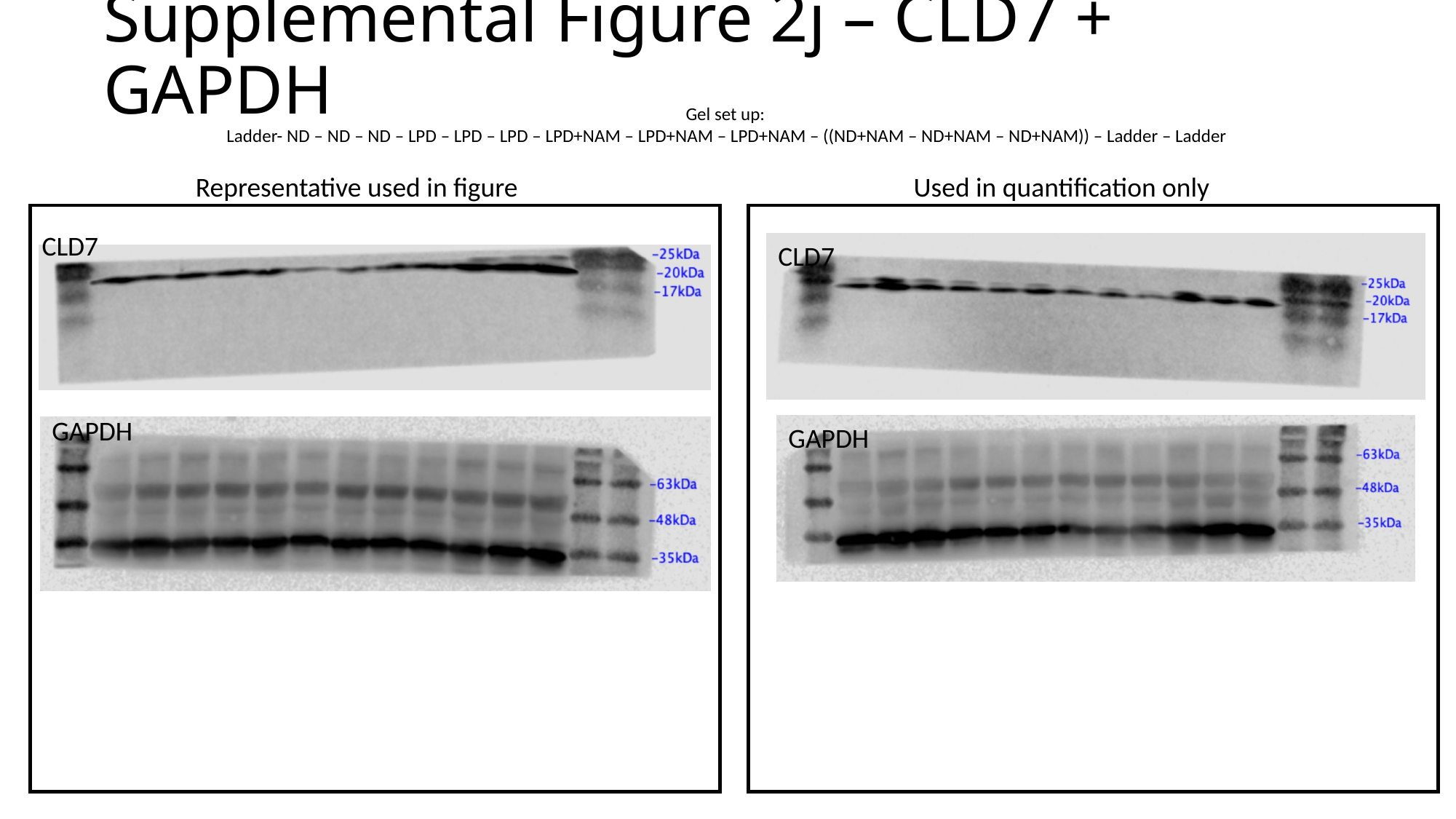

# Supplemental Figure 2j – CLD7 + GAPDH
Gel set up:
Ladder- ND – ND – ND – LPD – LPD – LPD – LPD+NAM – LPD+NAM – LPD+NAM – ((ND+NAM – ND+NAM – ND+NAM)) – Ladder – Ladder
Representative used in figure
Used in quantification only
CLD7
CLD7
GAPDH
GAPDH

## Slide 21
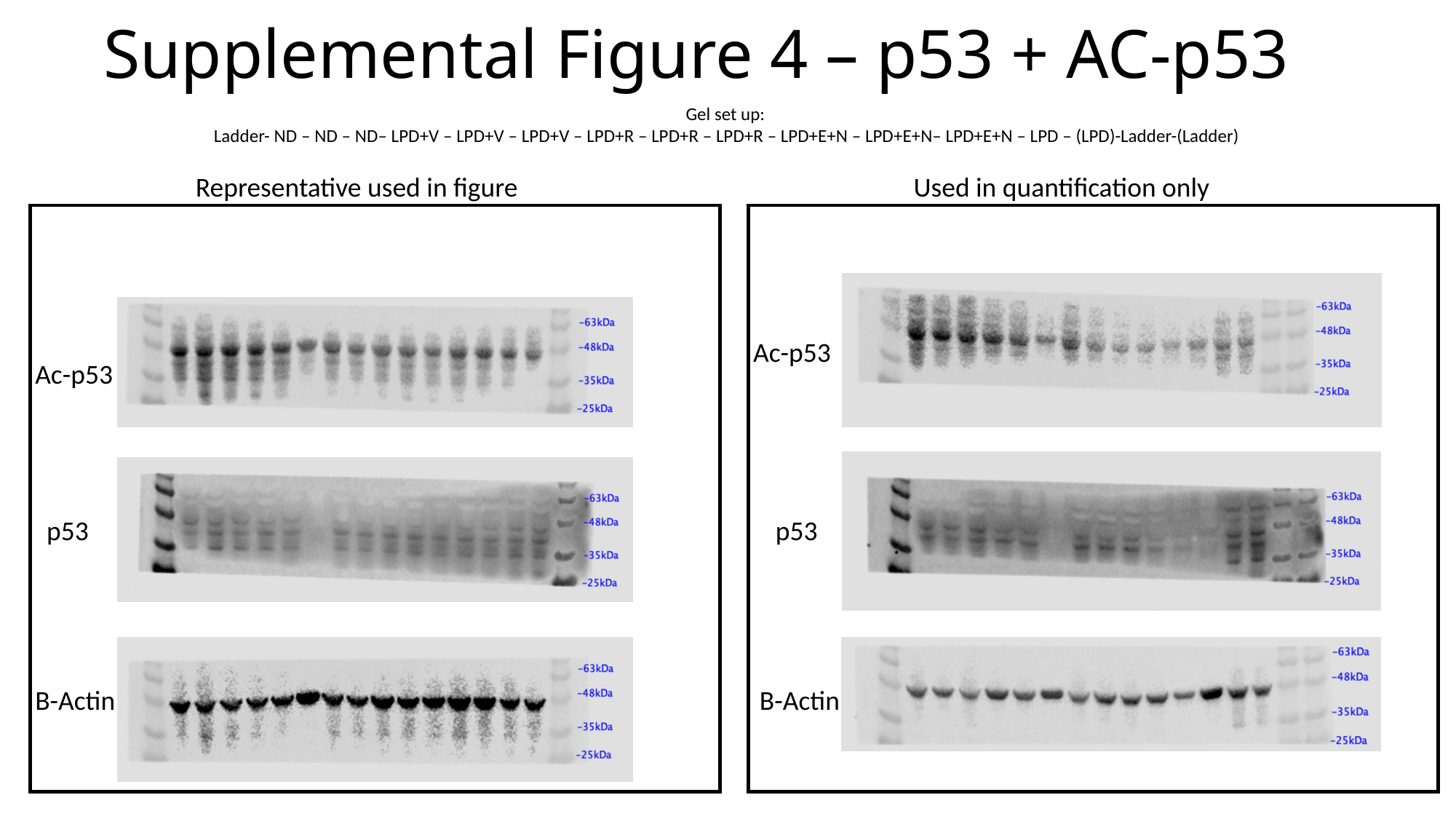

# Supplemental Figure 4 – p53 + AC-p53
Gel set up:
Ladder- ND – ND – ND– LPD+V – LPD+V – LPD+V – LPD+R – LPD+R – LPD+R – LPD+E+N – LPD+E+N– LPD+E+N – LPD – (LPD)-Ladder-(Ladder)
Representative used in figure
Used in quantification only
Ac-p53
Ac-p53
p53
p53
B-Actin
B-Actin
